# NS4/5 mutations enhance flavivirus Bamaga virus infectivity and pathogenicity *in vitro* and *in vivo*

**DOI:** 10.1371/journal.pntd.0008166

**Published:** 2020-03-23

**Authors:** Agathe M. G. Colmant, Helle Bielefeldt-Ohmann, Laura J. Vet, Caitlin A. O’Brien, Richard A. Bowen, Airn E. Hartwig, Steven Davis, Thisun B. H. Piyasena, Gervais Habarugira, Jessica J. Harrison, Jody Hobson-Peters, Roy A. Hall

**Affiliations:** 1 Australian Infectious Diseases Research Centre, School of Chemistry and Molecular Biosciences, The University of Queensland, St Lucia, Queensland, Australia; 2 School of Veterinary Science, The University of Queensland, Gatton, Queensland, Australia; 3 Department of Biomedical Sciences, Colorado State University, Fort Collins, Colorado, United States of America; 4 Berrimah Veterinary Laboratories, Department of Primary Industry and Resources, Northern Territory Government, Berrimah, NT, Australia; WRAIR, UNITED STATES

## Abstract

Flaviviruses such as yellow fever, dengue or Zika viruses are responsible for significant human and veterinary diseases worldwide. These viruses contain an RNA genome, prone to mutations, which enhances their potential to emerge as pathogens. Bamaga virus (BgV) is a mosquito-borne flavivirus in the yellow fever virus group that we have previously shown to be host-restricted in vertebrates and horizontally transmissible by *Culex* mosquitoes. Here, we aimed to characterise BgV host-restriction and to investigate the mechanisms involved. We showed that BgV could not replicate in a wide range of vertebrate cell lines and animal species. We determined that the mechanisms involved in BgV host-restriction were independent of the type-1 interferon response and RNAse L activity. Using a BgV infectious clone and two chimeric viruses generated as hybrids between BgV and West Nile virus, we demonstrated that BgV host-restriction occurred post-cell entry. Notably, BgV host-restriction was shown to be temperature-dependent, as BgV replicated in all vertebrate cell lines at 34°C but only in a subset at 37°C. Serial passaging of BgV in Vero cells resulted in adaptive mutants capable of efficient replication at 37°C. The identified mutations resulted in amino acid substitutions in NS4A-S124F, NS4B-N244K and NS5-G2C, all occurring close to a viral protease cleavage site (NS4A/2K and NS4B/NS5). These mutations were reverse engineered into infectious clones of BgV, which revealed that NS4B-N244K and NS5-G2C were sufficient to restore BgV replication in vertebrate cells at 37°C, while NS4A-S124F further increased replication efficiency. When these mutant viruses were injected into immunocompetent mice, alongside BgV and West Nile virus chimeras, infection and neurovirulence were enhanced as determined by clinical scores, seroconversion, micro-neutralisation, viremia, histopathology and immunohistochemistry, confirming the involvement of these residues in the attenuation of BgV. Our studies identify a new mechanism of host-restriction and attenuation of a mosquito-borne flavivirus.

## Author summary

Mosquito-borne pathogens include flaviviruses such as yellow fever virus, dengue virus and Zika virus, which continue to cause disease worldwide. Some of these flaviviruses have only recently emerged as major human pathogens, despite having been discovered decades ago. Determining the mechanisms of host-restriction of viruses with cryptic ecological niches will help us to understand how new viral diseases may emerge. In this study, we investigated the host-restriction of a recently discovered flavivirus, Bamaga virus. We demonstrated that the virus host-restriction observed in vertebrate cells only occurs at 37°C, and that the virus can replicate efficiently at lower temperatures. We identified three amino acid substitutions located at two viral protease cleavage sites, which we have demonstrated are directly involved in BgV host-restriction *in vitro* and attenuation *in vivo*. Overall, our data provide new insight into mechanisms flavivirus attenuation and host-restriction in vertebrates.

## Introduction

Flaviviruses have the capacity to emerge as major human pathogens and cause large scale epidemics. Recent examples include Zika and yellow fever viruses, which have caused large epidemics in South America in the 2010s, and dengue virus, which is still the most prevalent disease-causing arthropod-borne virus worldwide. While, most flaviviruses are transmitted through a classical arthropod-borne cycle between arthropod vectors and vertebrate hosts, and exhibit replication in cells from a wild range of invertebrate and vertebrate host species, some flaviviruses exhibit a more narrow host range. These include flaviviruses that do not infect arthropod hosts (no known vector), fail to replicate in vertebrates (insect-specific) or exhibit host-restriction in vertebrate cells that is cell line- or temperature-dependent [1–3]. The mechanisms for emergence of pathogens from enzootic flaviviruses are not clear despite past examples of large-scale epidemics caused by under-studied flaviviruses such as Zika virus [4]. The emergence of flaviviruses as pathogens may be linked to the naturally high rate of mutations in the genome of RNA viruses, due to the low proof-reading efficiency of the RNA-dependent RNA polymerase [5]. Indeed, flaviviruses are positive-sense, single-stranded RNA viruses with a genome of approximately 11 kilobases (kb), contained in an enveloped icosahedral nucleocapsid [6, 7]. The viral RNA contains an open reading frame (ORF) coding for a single polyprotein that is co- and post-translationally cleaved into three structural (capsid (C), membrane precursor (prM) / membrane (M) and envelope (E)), seven non-structural (NS) proteins and the 2K peptide (NS1, NS2A, NS2B, NS3, NS4A, 2K, NS4B, and NS5) [8, 9]. The translated polyprotein is embedded in the membranes of the endoplasmic reticulum *via* several transmembrane domains. It is cleaved by either a host signal peptidase in the endoplasmic reticulum lumen (C/prM, prM/E, E/NS1 and 2K/NS4B) or the viral protease NS2B-NS3 in the cytoplasm (NS2A/NS2B, NS2B/NS3, NS3/NS4A, NS4A/2K, NS4B/NS5). Once replication complexes have been established, with the NS5-encoded RdRp at their core, the viral RNA is replicated using a newly generated genome-length negative-sense strand as a template for new positive strands [10].

As part of our ongoing efforts to characterise flavivirus host-restriction, this study aimed to investigate Bamaga virus attenuation and the mechanisms involved. Bamaga virus (BgV) was recently isolated from archival mosquito samples of the *Culex sitiens* subgroup collected in 2001 and 2004 from Cape York, Far North Queensland, Australia [11] and found to be phylogenetically most closely related to Edge Hill virus and other members of the yellow fever group. Despite this close genetic relationship, initial *in vitro* characterisation showed that BgV displayed a restricted host range, as it was only able to replicate efficiently in a subset of vertebrate cell lines, and displayed a host-restricted phenotype in CD1 mice [11]. In an effort to classify BgV, its genome sequence was analysed for nucleotide composition and dinucleotide usage bias, which demonstrated that this virus most likely alternates between arthropod vectors and vertebrate hosts [12]. In addition, we recently reported that BgV could be horizontally transmitted by its only known vector, mosquitoes of the *Cx*. *sitiens* subgroup, since mosquitoes which were blood-fed with an infectious bloodmeal had infectious virus detected in their saliva after incubation, and could interfere with West Nile virus (WNV) and Murray Valley encephalitis virus replication *in vitro* and WNV transmission *in vivo* [13]. Since this host-restricted virus was shown to be capable of infecting some vertebrate cells and to be transmissible as part of a classical arbovirus transmission cycle, it is crucial to determine the factors of its apparent host-restriction. Indeed, identifying these restricting factors may help determine the likelihood for this virus to emerge as a major veterinary or human pathogen.

In this report, we have assessed the host-restriction of BgV in a wider range of cell lines and animal species. We have generated a BgV infectious clone and two chimeric viruses between BgV and WNV to investigate which BgV genes might be responsible for its host-restriction both *in vitro* and *in vivo*. We investigated the effect of temperature on BgV host-restriction *in vitro* and then passaged the virus in vertebrate cells at various temperatures, which revealed that BgV host-restriction and attenuation were influenced by specific amino acid substitutions in the viral non-structural proteins. Uncovering the mechanisms involved in the host-restriction and attenuation of flaviviruses is crucial to understanding the mechanisms for emergence of new flaviviral diseases and to designing strategies for the development of vaccines and therapeutics against pathogenic flaviviruses.

## Results

### BgV is host-restricted in some vertebrate cells *in vitro*

We have previously shown that BgV replication could be detected in OK, HEK293 and BHK cells but not in Vero, DF-1, or RK-13 cells [11]. BgV replication was assessed in a wider range of insect and vertebrate cell lines at 28°C or 37°C, including in cell lines used in our previous study, as shown in Table 1. In insect cells (C6/36, MOS55, S2), BgV replicated as well as or better than the WNV_KUN_ reference virus. BgV also replicated in some vertebrate cell lines at 37°C (MDCK, PK15, LLC PK1, ST, OK, BHK, BSR, HEK293 and SW13) or 28°C (VSW, 3CPL), to a similar or lower titre than WNV_KUN_. However, a subset of vertebrate cells (Vero, DF-1, RK-13, MEF_WT_, JU56), did not support BgV replication at 37°C, and another subset also failed to enable BgV to replicate at 28°C (A6, 3CPK, 1LV), despite supporting WNV_KUN_ replication at these temperatures.

**Table 1:** Replication of BgV in insects and vertebrates. Insect and vertebrate cell lines were inoculated in triplicates with BgV and WNV at MOI 1 for 1h 30min, the inoculum removed and the cells washed before incubating the replenished cultures at 28°C or 37°C. The inoculated cell culture supernatants were harvested five days p.i., titrated on C6/36 cells and analysed by fixed-cell ELISA.—represents no replication, + represents a titre over 10^2^ TCID_50_IU/mL, ++ represents a titre over 10^4^ TCID_50_IU/mL, +++ represents a titre over 10^6^ TCID_50_IU/mL. In a few instances, not all replicates were positive, in which case the titre represented is the average of the positive replicates only.

Cell line		Temperature	Replication levels	
Animal	Name		BgV	WNV_KUN_
Mosquito	C6/36*	28°C	+++	+++
	HSU		-	-
	MOS55		+++	++
Drosophila	S2		++	++
Viper	VSW	28°C	+ˠ	++
Crocodile	3CPL		+ˠ	++
Dog	MDCK	37°C	+	++
Pig	PK15		+ˠ	++
	LLCPK1		+	++
	ST		++	+++
Opossum	OK*		++	++
Hamster	BHK*		++	+++
	BSR		+++	+++
Human	HEK293*		+	+++
	SW13		+++	+++
Frog	A6	28°C	-	+
Crocodile	3CPK		-	++
	1LV		-	++
Monkey	Vero*	37°C	-	+++
Chicken	DF-1*		-	+++
Rabbit	RK-13*		-	++
Cow	MDBK		-	-
Mouse	MEF_WT_		-	+++
	MEF_IFNAR-/-_		-	+++
	MEF_RNL-/-_		-	+++
Wallaby	JU56		-	+ˠ

- < limit of detection (10^2^ TCID_50_IU/mL);

+ > 10^2^ TCID_50_IU/mL; ++ > 10^4^ TCID_50_IU/mL; +++ > 10^6^ TCID_50_IU/mL

ˠ Not all replicates were positive

*BgV host-restriction in these cell lines was published previously in Colmant et al. 2016. The results presented here are from repeat experiments in these cell lines.

### Sensitivity to interferon or RNAse L responses is not determinant in BgV host-restriction *in vitro*

Two knockout strains of MEF cells were included among the cell lines tested for BgV replication in an attempt to elucidate whether BgV host-restriction was due to the anti-flaviviral activity from RNAse L or to the antagonistic effect of an IFN-I response. Neither MEF_RNL-/-_, which lack RNAse L, nor MEF_IFNAR-/-_, which lack receptors for type I interferons (IFN-I), supported BgV replication, suggesting that these mechanisms were not determinant (Table 1).

### BgV is host-restricted in vertebrates *in vivo*

BgV was inoculated into various animal species to confirm its host-restricted phenotype and to complement the mouse infection data obtained previously [11]. Embryonated chicken eggs have been used extensively to culture and isolate flaviviruses and are susceptible to infection with a wide range of flaviviruses [14]. Injecting 9–12 days old embryonated eggs intravenously with BgV did not produce any visible disease except for one embryo which received 10^4.7^ TCID_50_ infectious units (IU) and died 120h post-injection. BgV was not detected in the homogenate of the three embryos inoculated with the lowest dose (10^3.7^ TCID_50_ IU/egg) or in the embryo that died. Only low levels of virus (10^4.1–4.9^ TCID_50_ IU/egg) were detected in the remaining homogenates, most likely due to residual virus from the injections (Table 2). BgV was also inoculated subcutaneously into avian, reptilian and amphibian animal models, including five house sparrows (*Passer domesticus*), six northern leopard frogs (*Lithobates pipiens*), six Texas toads (*Anaxyrus speciosus*), and six ribbon snakes (*Thamnophis saurita*). All animals were sampled for blood at multiple time points post-infection (p.i.) (Table 2). No infectious virus was detected in any serum sample at any time point. Taken together, these data show that BgV replication was not detected in avian, reptilian or amphibian models.

**Table 2:** BgV replication in vertebrate animal models. Groups of three eggs were inoculated with three doses of BgV intravenously (10^5.7^, 10^4.7^ and 10^3.7^ TCID_50_ IU/egg, respectively), incubated at 33–35°C for five days and homogenised. The homogenates were titrated on C6/36 cells and the resulting virus titres were analysed by fixed-cell ELISA. The eggs injected at the higher doses had traces of virus present but at levels suggesting they resulted from leftover inoculum rather than virus replication. The other animal models were inoculated with 10^6^ TCID_50_ IU of BgV and sampled for blood on days 1–5 and 14 post-inoculation (sparrows) or on days 1, 3, 5, 7, and 28 post-inoculation (frogs, toads, snakes). The blood samples were diluted 1:5 and 1:50 and inoculated on C6/36 cells which were incubated for two days and analysed for virus by fixed-cell ELISA.

Species	Dose (TCID_50_ IU)	BgV positive / tested							
		Day 1	Day 2	Day 3	Day 4	Day 5	Day 7	Day 14	Day 28
*Gallus gallus–* Embryonated chicken eggs	10^5.7^	*NT*	*NT*	*NT*	*NT*	0 / 3 *	*NT*	*NT*	*NT*
	10^4.7^	*NT*	*NT*	*NT*	*NT*	0 / 3 *	*NT*	*NT*	*NT*
	10^3.7^	*NT*	*NT*	*NT*	*NT*	0 / 3	*NT*	*NT*	*NT*
*Passer domesticus–* House sparrow	10^6.0^	0 / 5	0 / 5	0 / 5	0 / 5	0 / 5	*NT*	0 / 5	*NT*
*Lithobates pipiens–* Northern leopard frog	10^6.0^	0 / 6	*NT*	0 / 6	*NT*	0 / 6	0 / 6	*NT*	0 / 6
*Anaxyrus speciosus–* Texas toad	10^6.0^	0 / 6	*NT*	0 / 6	*NT*	0 / 6	0 / 6	*NT*	0 / 6
*Thamnophis saurita–* Ribbon snake	10^6.0^	0 / 6	*NT*	0 / 6	*NT*	0 / 6	0 / 6	*NT*	0 / 6

IU: infectious units; NT–not tested.

* BgV was detected in eggs injected at higher doses, at levels suggesting no replication took place but residual inoculum was present.

### Production of infectious DNAs encoding the prototype BgV genome or chimeric BgV-WNV genomes

**Fig 1:**
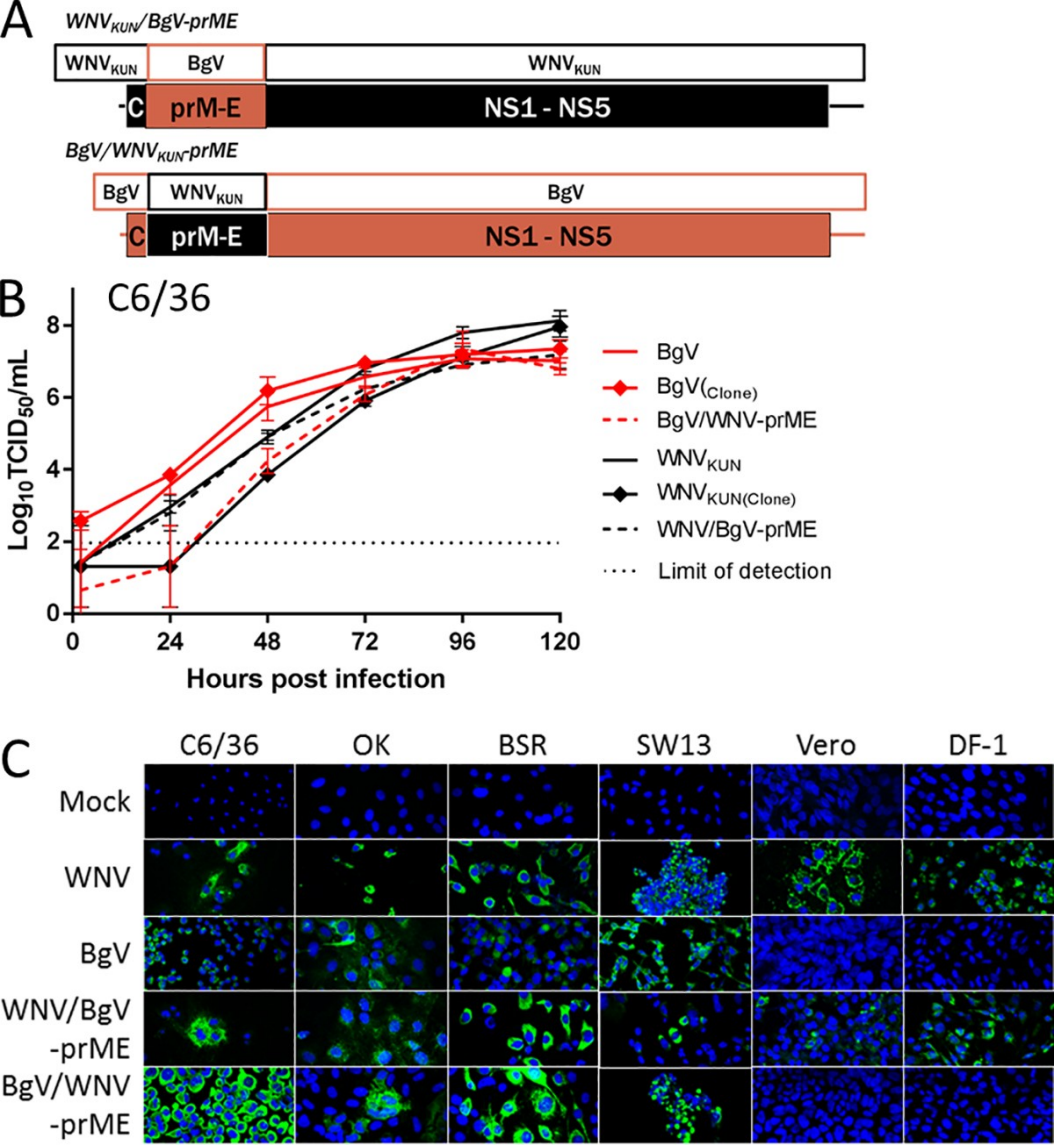
A. Schematic representation of the genome composition of CPER-generated BgV-derived chimeric viruses. Black shading indicates WNV_KUN_ genes while red shading indicates BgV genes. The prME genes were swapped for each virus, generating a chimera with BgV “backbone” sequence and WNV_KUN_ prME: BgV/WNV-prME and the reverse chimera with WNV backbone sequence and BgV prME: WNV/BgV-prME. B. Replication kinetics of parental prototype viruses and CPER-generated chimeras and infectious clones in C6/36 cells over five days. The insect cells were inoculated with each virus at MOI 0.1 in triplicates for 1h 30min, the inoculum removed and the cells washed before incubating the replenished cultures at 28°C. The supernatants were harvested at 2, 24, 48, 72, 96 and 120 hours p.i., stored at -80°C until they were titrated on C6/36 cells and the titres determined by fixed-cell ELISA. Error bars represent the standard deviation and the dotted lines represent the lower limit of detection. C. Visualisation of virus replication in vertebrate cells by IFA. The cells were inoculated at MOI 1 with WNV, BgV, WNV/BgV-prME, BgV/WNV-prME or mock inoculated, in triplicates, the inoculum removed and the cells washed before incubating at 37°C (all vertebrate cells) or 28°C (C6/36 cells). The cells were fixed five days post-inoculation and stained with pan-flavivirus mAb 4G2 by IFA.

To identify the viral factors associated with BgV host-restriction, we constructed infectious DNAs of BgV as well as chimeric viruses with a genome that contained genes of both BgV and a more promiscuous flavivirus, WNV. The BgV full genome sequence was first completed by sequencing the extreme ends of the BgV UTRs using RNA circularisation [15, 16] (Genbank accession numbers: 5’ UTR MH257543, 3’ UTR MH257544). With this information, circular polymerase extension reaction (CPER) [17] was used to generate infectious cDNA of BgV and two chimeric constructs (Fig 1A). One chimera contained the WNV_KUN_ genome as a backbone sequence with its prME genes replaced with those from BgV (WNV/BgV-prME); the other contained the BgV genome as a backbone with its prME genes switched for those from WNV_KUN_ (BgV/WNV-prME) (Fig 1A). The C/prM and E/NS1 junctions used to design the chimeras followed the WNV_KUN_ and BgV published annotations. Rescued viruses were passaged in C6/36 cells and their full genomes sequenced and compared to the expected sequences based on their parental viruses. The only amino acid difference observed was a substitution in BgV/WNV-prME NS1-E342G (nucleotide mutation A3338G). All infectious DNA-derived viruses (BgV, WNV_KUN_, WNV/BgV-prME, BgV/WNV-prME) were compared with native isolates of BgV and WNV_KUN_ in a replication kinetics assay in C6/36 cells. BgV/WNV-prME displayed significantly slower replication than BgV at early time points (*p* = 0.0001 at 24h and *p* = 0.0254 at 48h) and so did the WNV infectious clone-derived virus compare to the WNV isolate (*p* = 0.0101 at 24h). Aside from these two instances, all CPER-derived viruses displayed similar replication kinetics in insect cells compared to their parental virus (Fig 1B).

### BgV host-restriction likely occurs post-entry *in vitro*

To identify the viral factors associated with BgV host-restriction *in vitro*, we assessed the ability of the chimeric viruses (WNV/BgV-prME and BgV/WNV-prME) to replicate in a selection of vertebrate cells (Fig 1C). These cell lines were chosen as representatives of BgV-permissive and BgV-non-permissive cell lines. Examination of inoculated cell monolayers by IFA revealed that similar to WNV_KUN_, WNV/BgV-prME could replicate in all tested vertebrate cell lines, while BgV/WNV-prME could only replicate in OK, BSR and SW13 cells, similar to BgV (Fig 1C). This demonstrated that BgV host-restriction was associated with the non-structural proteins or UTRs of the virus, and likely occurred post-entry to the cells.

### BgV host-restriction *in vitro* is temperature-dependent

**Fig 2:**
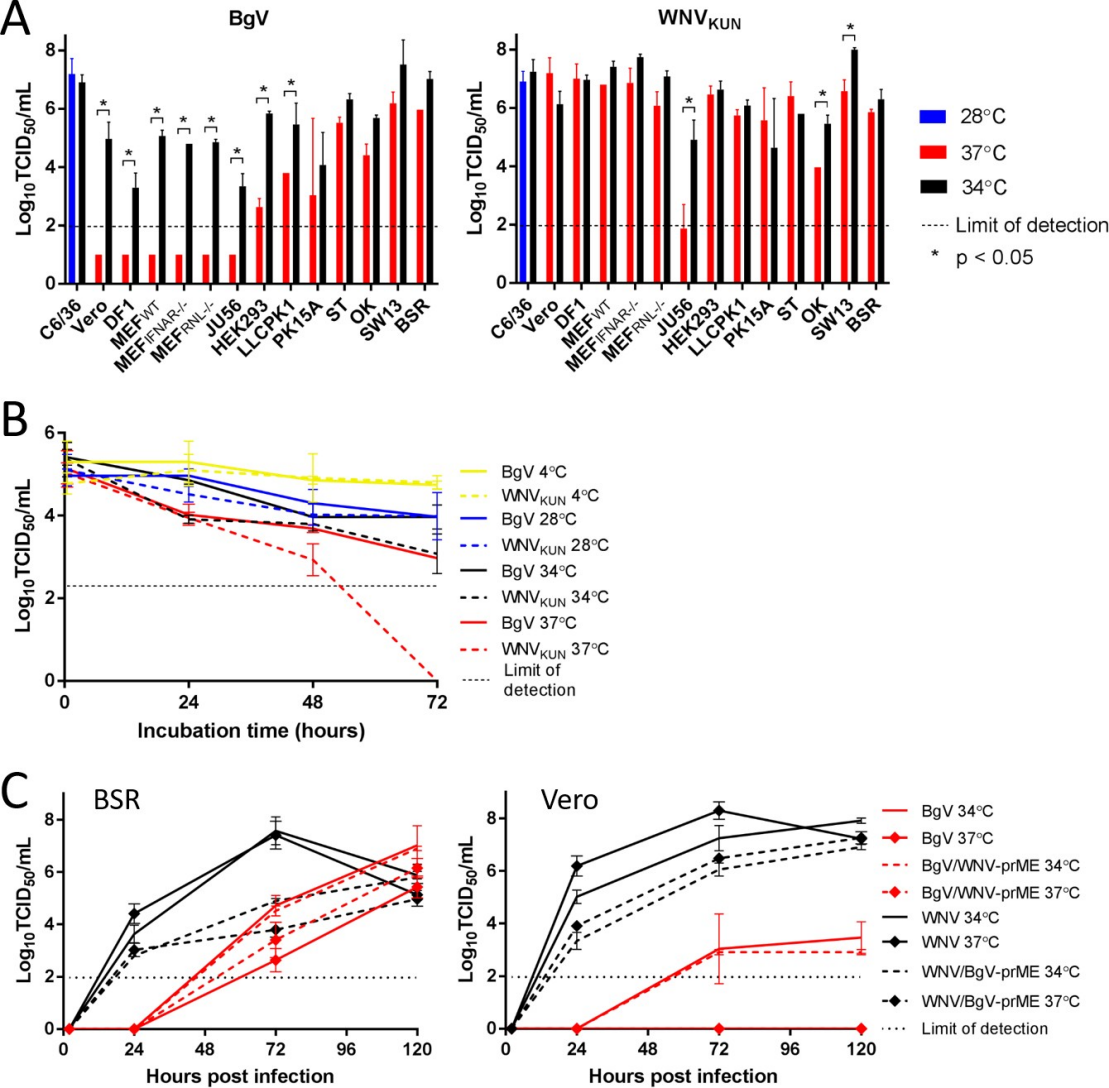
A. Replication of BgV and WNV_KUN_ in vertebrate cells. Cells were inoculated at MOI 1 with BgV or WNV_KUN_ in triplicate for 1h 30min, the inoculum removed and the cells washed before incubating at 34°C (black), 37°C (red) or 28°C (blue) with fresh medium. The inoculated cell culture supernatants were harvested five days p.i., titrated on C6/36 cells and analysed by fixed-cell ELISA. The dotted line represents the lower limit of detection, * stands for *p* < 0.05. Error bars represent the standard deviation. B. Thermostability of BgV and WNV_KUN_ at various temperatures over three days. Triplicates of 10^5^ TCID_50_ IU of BgV and WNV_KUN_ were held at 4°C, 28°C, 34°C and 37°C in culture medium with 2% FBS in the absence of cells, harvested at 30min, 24h, 48h and 72h after the start of incubation and stored at -80°C. These samples were titrated on C6/36 cells and the titres determined by fixed-cell ELISA. Error bars represent the standard deviation. C. Replication kinetics of BgV, BgV/WNV-prME, WNV_KUN_ and WNV/BgV-prME, in BSR (left) and Vero cells (right) over five days at 34°C and 37°C. The cells were inoculated at MOI 0.1 in triplicates at 34°C or 37°C for 1h 30min, the inoculum removed and the cells washed before incubating at either temperature with fresh medium. The supernatants were harvested at 2, 24, 72 and 120 hours p.i., stored at -80°C until they were titrated on C6/36 cells and the titres determined by fixed-cell ELISA. Error bars represent the standard deviation and the dotted lines represent the lower limit of detection.

In order to further define BgV host-restriction, a subset of the vertebrate cell lines tested in Table 1 was inoculated with BgV and incubated at 34°C, instead of the usual 37°C. Notably, BgV was found to replicate at 34°C in all vertebrate cell lines tested, including those not supporting its replication at 37°C (Fig 2A). Using a thermal stability assay, we determined that this temperature-dependent host-restriction was not due to the virus being less stable at higher temperatures. Indeed, when BgV and WNV_KUN_ were held at various temperatures in the absence of cells over 72 hours, the infectivity of BgV was as stable or better than that of WNV_KUN_ at all temperatures tested (4°C, 28°C, 34°C and 37°C) (Fig 2B). We determined a threshold of temperature for this phenotype, by passaging a C6/36-derived BgV stock onto Vero (non-permissive for BgV at 37°C) and BSR cells (permissive for BgV at 37°C) at 34°C, 35°C, 36°C and 37°C. This revealed that BgV was able to replicate in both cell lines at 34°C (10^5.8^ TCID_50_ IU/mL in both), 35°C (10^5.1^ TCID_50_ IU/mL in Vero cells, 10^6.9^ TCID_50_ IU/mL in BSR cells) and 36°C (10^3.1^ TCID_50_ IU/mL in Vero cells, 10^4.7^ TCID_50_ IU/mL in BSR cells) but only in BSR cells at 37°C (10^3.9^ TCID_50_ IU/mL).

In order to elucidate whether BgV temperature-dependent host-restriction was associated with the structural or non-structural genes of BgV, a replication kinetics assay was performed with BgV, WNV_KUN_, WNV/BgV-prME and BgV/WNV-prME in BSR and Vero cells at 34°C and 37°C. BgV/WNV-prME and BgV replicated at 34°C and 37°C in BSR cells, with more robust growth at 34°C, but failed to produce detectable levels of progeny virus in Vero cells at 37°C (Fig 2C). In contrast, WNV/BgV-prME and WNV_KUN_ were able to replicate in BSR and Vero cells at both temperatures, although WNV/BgV-prME reached lower peak titres than WNV_KUN_ and had no discernible cytopathic effect on the cells (Fig 2C). This demonstrated that the temperature-dependent host-restriction of BgV was associated with the non-structural proteins or UTRs of this virus.

### BgV quickly acquires replicative competence in vertebrate cells at 37°C upon passaging at increasing temperatures

**Fig 3:**
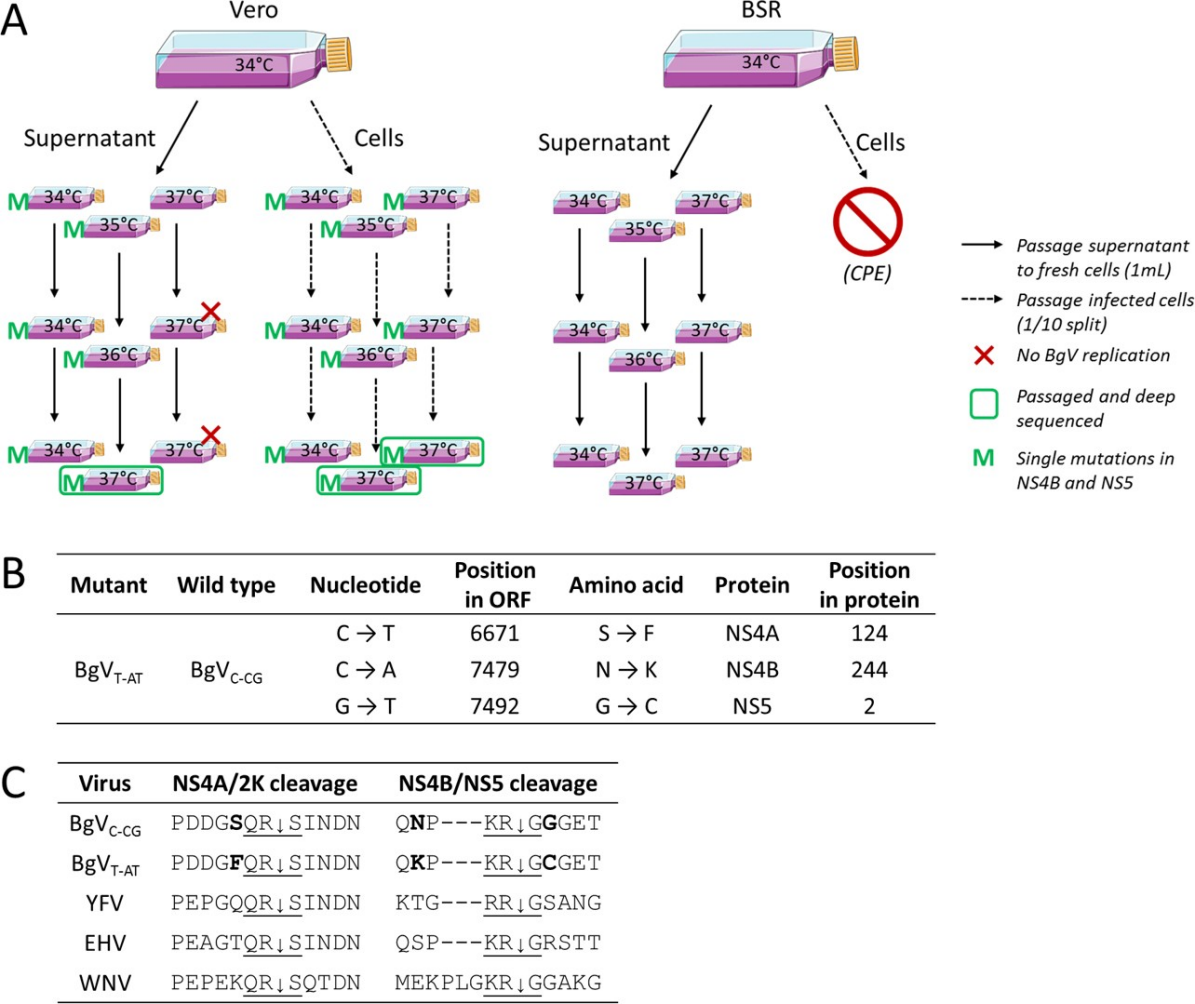
A. Blind passages of BgV in vertebrate cells. Flasks of BSR and Vero cells were inoculated with BgV at MOI 1 and incubated at 34°C for five days. 1mL aliquots of supernatant from these flasks were blindly passaged three times to flasks of freshly seeded cells. Vero cells were split 1/10 and passaged three times, while BSR cells succumbed to cytopathic effect and could not be passaged. At each passage, one set of supernatant and cells was maintained at either 34°C or 37°C, while the third set was incubated at increasing temperatures: 35°C, 36°C and finally 37°C. The presence of BgV in the harvested supernatants was tested by titration on C6/36 cells followed by fixed-cell ELISA. The red crosses represent samples in which BgV was not detected. Three samples were selected for further passaging (circled) and RNA extracts from the resulting supernatants were deep sequenced. The samples marked with a green M carried two of three identified conserved mutations, as determined by Sanger sequencing, in NS4B and NS5. B. The three conserved mutations identified by next-generation sequencing in replication-competent BgV variants. The nomenclature adopted hereafter is BgV_C-CG_ for prototype BgV and BgV_T-AT_ for a BgV variant containing all three NS4A/NS4B/NS5 identified conserved mutations. C. Alignment of selected flaviviruses over two viral protease cleavage sites: NS4A/2K and NS4B/NS5. The bolded letters correspond to the identified mutation sites in BgV sequence. The underlined letters and downward arrow correspond to the viral protease cleavage site.

BgV was passaged in BSR and Vero cultures in an attempt to select for BgV variants which would grow in all vertebrate cells at 37°C. At each passage, 1mL of supernatant was blindly transferred onto fresh uninfected cells, while the infected cells were split 1 in 10 with fresh medium (Fig 3A). Each culture was incubated at temperatures between 34°C and 37°C (see Fig 3A). When we analysed the supernatants from all passaged cultures for the presence of BgV, we found that replication-competent BgV variants had quickly arisen in the majority of Vero-derived samples, with only two BgV-negative samples (red crosses in Fig 3A). Three selected supernatants (circled in green in Fig 3A) were passaged once more on both BSR and Vero cells at 37°C and RNA extracted from the resulting supernatant was deep sequenced alongside the original inoculum. This revealed three nucleotide mutations (C6671T; C7479A; G7492T) corresponding to three amino acid changes (NS4A-S124F; NS4B-N244K; NS5-G2C) in the replication-competent variants that were not present in the original inoculum (Fig 3B). When viral RNA from all harvested supernatants were sequenced across the regions of these three sites, it was revealed that C7479A and G7492T were quickly selected for, even in cultures held at 34°C, and were always found concomitantly (green M in Fig 3A). C6671T was always found in combination with the other two mutations as a triple mutation and only in some Vero-derived deep sequenced samples, cultured at 37°C. Based on these results, the following nomenclature was adopted: BgV_X-YZ_ referring to the nucleotide in position 6671 (X), 7479 (Y) and 7492 (Z), with BgV_C-CG_ corresponding to the prototype virus and BgV_T-AT_ corresponding to a variant with all three sites mutated (Fig 3B). The three mutations were found to be in close proximity to a viral protease cleavage site: NS4A-S124F is the amino acid prior to the NS4A/2K cleavage site, and NS4B-N244K and NS5-G2C are in positions -2 and +1 respectively compared to the NS4B/NS5 cleavage site (Fig 3C).

### Reverse engineered mutants can replicate in Vero and DF-1 cells at 37°C

**Fig 4:**
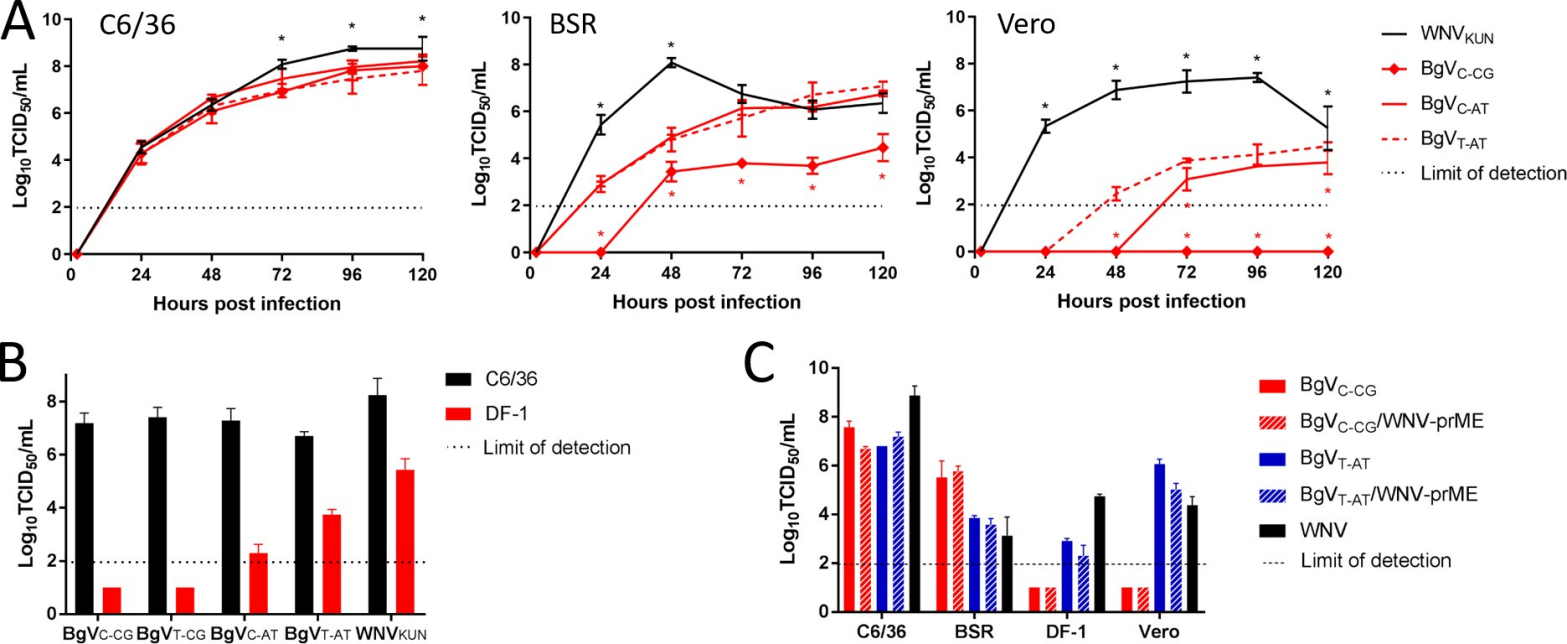
A. Replication kinetics of BgV_C-CG_ (prototype), BgV_C-AT_ (double mutant NS4B/5), BgV_T-AT_ (triple mutant NS4A/4B/5) and WNV_KUN_ in C6/36 cells (28°C—left), BSR cells (37°C—middle) and Vero cells (37°C—right) over five days. The cells were inoculated with each virus at MOI 0.1 in triplicate, incubated 1h 30min at 28°C or 37°C, the inoculum removed and the cells washed before incubating at 28°C or 37°C with fresh medium. The supernatants were harvested at 2, 24, 48, 72, 96 and 120 hours p.i., stored at -80°C until they were titrated on C6/36 cells and the titres determined by fixed cell ELISA. Error bars represent the standard deviation and the dotted lines represent the lower limit of detection. The graph includes only representations of statistical analysis for comparisons to BgV_T-AT_ for clarity (* = *p* < 0.05). The results of the comparisons to BgV_C-AT_ were the same as those to BgV_T-AT._ A star can therefore be read as a statistically significant difference to the mutants at that time point. B. BgV variants and WNV_KUN_ replication in DF-1 cells at 37°C. The cells were inoculated with each virus in triplicate at MOI 1 and incubated at 28°C (C6/36) or 37°C (DF-1). The supernatants were harvested after five days, titrated on C6/36 cells and the titres determined by fixed cell ELISA. The error bars represent the standard deviation and the dotted line represents the lower limit of detection. C. Prototype BgV_C-CG_, BgV_C-CG_/WNV-prME, and mutant BgV_T-AT_, BgV_T-AT_/WNV-prME and WNV_KUN_ replication in vertebrate cells at 37°C. Each cell line was inoculated with each virus in triplicate at MOI 1 as above and incubated at 28°C (C6/36) or 37°C (BSR, DF-1, Vero). The supernatants were harvested, titrated on C6/36 cells and the titres determined by fixed-cell ELISA. Error bars represent the standard deviation and the dotted line represents the lower limit of detection.

Three BgV variants were generated (BgV_T-CG_, BgV_C-AT_ and BgV_T-AT_) alongside a prototype BgV (BgV_C-CG_) in order to confirm the effect of the mutations on BgV replication in vertebrate cells. The mutation site sequences were confirmed in rescued viruses passaged once on C6/36 cells. The host-restriction and growth kinetics of these mutants were assessed in C6/36 cells at 28°C and in BSR and Vero cells at 37°C (Fig 4A, S1 Fig). These assays showed that all BgV variants and prototype BgV_C-CG_ had similar replication kinetics in C6/36 cells. The prototype BgV_C-CG_ and the NS4A single mutant BgV_T-CG_ displayed similar growth patterns on BSR and Vero cells, with no replication detected in Vero cells, but efficient replication detected in BSR cells, with faster replication for the mutant compared to the prototype virus in the latter (S1 Fig). The double and triple mutants BgV_C-AT_ and BgV_T-AT_ replicated faster and to higher titres than prototype BgV_C-CG_ in both vertebrate cell lines, with a slight advantage for the triple mutant BgV_T-AT_ in Vero cells (Fig 4A). The peak titres for all Bamaga virus variants were still lower than for the reference virus WNV_KUN_ in both vertebrate cell lines (Fig 4A).

We also assessed the replication efficiency of the mutant BgV viruses in DF-1 cells at 37°C to determine whether the identified mutations rescued BgV replication in cell lines other than Vero cells. As expected, prototype BgV_C-CG_ and NS4A mutant BgV_T-CG_ replication was not detected, while double and triple mutants BgV_C-AT_ and BgV_T-AT_ replicated in DF-1 cells at 37°C, with an advantage for BgV_T-AT_ compared to BgV_C-AT_ (Fig 4B).

BgV_T-AT_ was then used as a template to generate a mutated version of the prototype BgV_C-CG_/WNV-prME chimera: BgV_T-AT_/WNV-prME, which was confirmed to replicate in C6/36 cells with similar efficiency to prototype BgV_C-CG_ and BgV_C-CG_/WNV-prME and to mutant BgV_T-AT_ after 5 days (Fig 4C). This mutant chimera was created in an attempt to remove multiple BgV host-restriction factors simultaneously. Indeed, WNV_KUN_ prM-E proteins should provide efficient entry to the cells (Fig 2), while the three NS4/5 substitutions should ablate the post-entry restriction at 37°C. As expected, the mutated chimera replicated in all tested vertebrate cells (BSR, DF-1 and Vero) at 37°C to similar or slightly lower titres than mutant BgV_T-AT_ (Fig 4C). While these *in vitro* experiments did not show an enhanced replication of BgV_T-AT_/WNV-prME compared to BgV_T-AT_ in the cell lines tested, further studies were required to determine if this phenotype was representative of an *in vivo* infection.

### Virus replication and pathogenesis *in vivo* is enhanced by NS4/5 substitutions identified *in vitro*

The host-restriction of BgV was further assessed *in vivo*, by inoculating 19-days old immunocompetent CD-1 mice intracranially (IC) and intraperitoneally (IP) with 10^4^ TCID_50_ IU of prototype and mutant BgV (BgV_C-CG_, n = 31 and BgV_T-AT_, n = 12 respectively), prototype and mutant chimeras (BgV_C-CG_/WNV-prME, n = 12 and BgV_T-AT_/WNV-prME, n = 12) and the reverse chimera (WNV/BgV-pME, n = 20). Subsets of mice were terminated on days three and five p.i., respectively, and subjected to blood and tissue sampling for virus isolation and histopathology. The remaining mice were culled on day 21–22 p.i. and similarly subjected to blood and tissue sampling. The serum samples were used in two assays: fixed-cell ELISA and microneutralisation, to determine the levels of virus antigen-reactive and neutralising antibodies, respectively.

**Fig 5:**
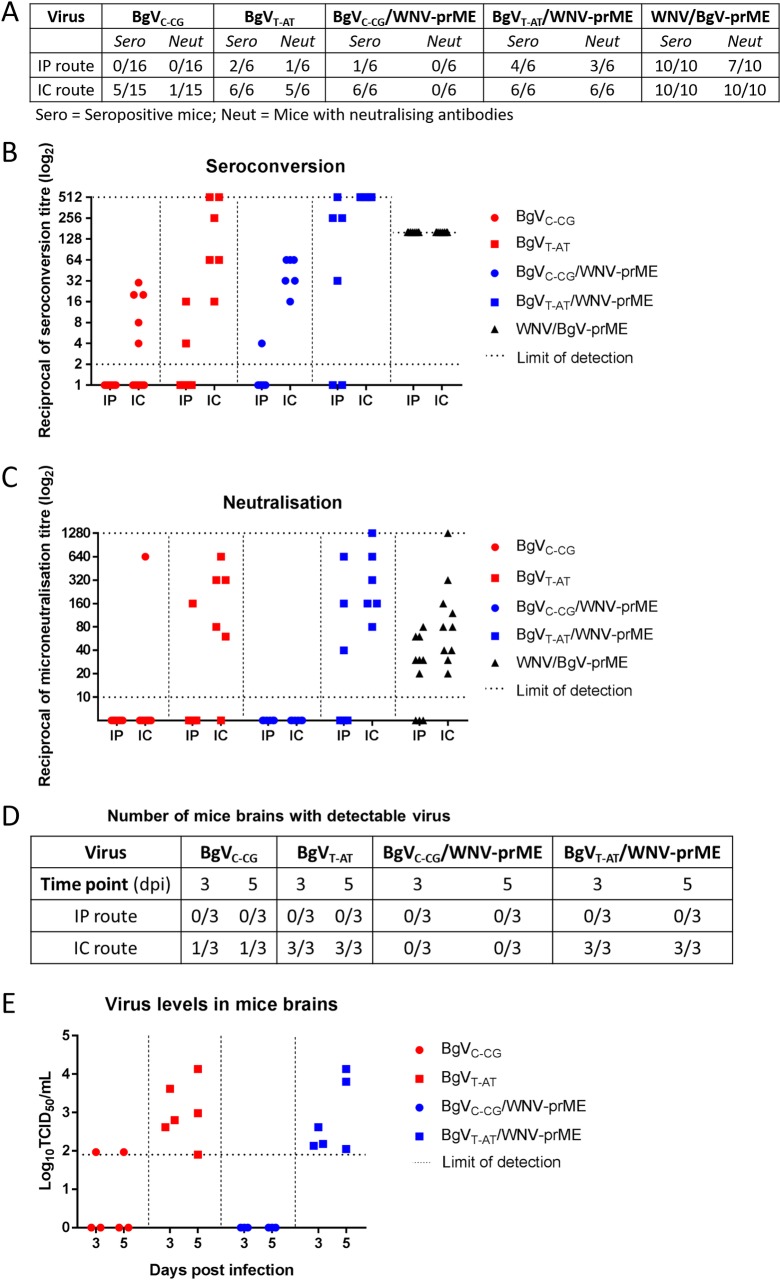
A. Summary of seropositive mice inoculated with BgV-derived viruses and mice with neutralising antibodies. Seroconversion was determined by fixed-cell ELISA using doubling dilutions of sera on BgV infected and fixed C6/36 cells. B. Levels of seroconversion obtained from BgV-derived viruses-injected mice. The dotted lines represent the lower and higher limits of detection. Sera from WNV/BgV-prME-injected mice were only diluted to 1/160 as opposed to the rest of the sera, diluted up to 1/512. The sample size of each group can be found in (A). C. Levels of neutralising antibodies in BgV-derived viruses-injected mice sera as determined by microneutralisation assay. The dotted lines represent the lower and higher limit of detection. The sample size of each group can be found in (A). D. Summary of virus positive mice brains three and 5 five days p.i.. The left brain hemisphere was harvested from three mice per time point in each group, stored at -80°C, weighed, homogenised in a 20% w/v mixture with RPMI, titrated on C6/36 cells and analysed by fixed-cell ELISA. E. Levels of detected virus in mice brains as determined by titration on C6/36 cells, see protocol in (D). The dotted line represents the lower limit of detection.

First, the prototype BgV_C-CG_ was confirmed to have the same mild phenotype as previously reported [11]. None of the mice developed any clinical signs of disease at any stage after inoculation. We detected no seroconversion in IP-injected mice, suggesting negligible replication in peripheral tissues, but 33% of IC-injected mice had seroconverted or developed neutralising antibodies, suggesting some replication occurred in neural tissues in these mice (Fig 5A–5C).

By comparison, the mutant BgV_T-AT_ replicated in 33% of the IP-injected mice and in 100% of the IC-injected mice (Fig 5A–5C). The antibody titres in seropositive IC-injected mice were higher on average for BgV_T-AT_ than for prototype BgV_C-CG_ (Fig 5B). One BgV_T-AT_ IC-injected mouse developed mild clinical signs (growth retardation, bloated abdomen, ruffled coat, hunching). It was included in the scheduled day 5 p.i. cohort cull for harvest of organs for virus isolation (see below). Necropsy revealed a gastro-intestinal tract full of gas and an under-developed spleen. Together these data show that the NS4/5 substitutions in BgV_T-AT_ enhance infectivity and pathogenicity in mice compared to prototype BgV_C-CG_.

Additional groups were IP- and IC-inoculated with prototype or mutant BgV chimeras. Protoype BgV_C-CG_/WNV-prME replicated in 17% of IP-inoculated mice and in all IC-inoculated mice as shown by seroconversion (Fig 5A and 5B), however, no clinical signs of disease and no neutralising antibodies were recorded for any BgV_C-CG_/WNV-prME-inoculated mice (Fig 5A–5C). Nevertheless, the ability of BgV_C-CG_/WNV-prME to establish an infection is enhanced when compared to BgV_C-CG_.

The mutant BgV_T-AT_/WNV-prME replicated in 67% IP-inoculated mice and all IC-inoculated mice, suggesting more efficient replication in peripheral tissues (Fig 5A–5C). In addition, one BgV_T-AT_/WNV-prME IC-inoculated mouse displayed intermittent clinical signs of disease including mild depression of activity, growth retardation and a weak grip between days 9 and 17 p.i. but never reached a score requiring euthanasia and survived until 22 days p.i.. Thus, mutant BgV_T-AT_/WNV-prME displays enhanced infectivity and pathogenicity in mice compared to BgV_C-CG_/WNV-prME, due to the introduction of the NS4/5 substitutions. The mutant chimera also replicated more efficiently than mutant BgV_T-AT_ *in vivo*.

The reverse chimera WNV/BgV-prME replicated efficiently in both IP- and IC-inoculated mice, with all mice seroconverting (Fig 5A–5C). One mouse IC-injected with WNV/BgV-prME displayed clinical signs of neurological disease (hunching, very restricted mobility, very ruffled coat) and was culled at its humane endpoint, 8 days p.i..

Mice from groups infected with the prototype and mutant BgV and BgV/WNV-prME chimeras were culled at three and five days p.i. and sampled for virus isolation (serum, brain, spleen, kidney, liver, mesenteric lymph node) in an attempt to characterise BgV viremia and replication *in vivo*. Low levels of infectious virus were detected in the brains of only 33% of prototype BgV_C-CG_ IC-inoculated mice, while virus was re-isolated from all brains from mutant BgV_T-AT_ and BgV_T-AT_/WNV-prME IC-inoculated mice at both time points (Fig 5D and 5E). The virus titres were not significantly different between viruses at each time point (*p* > 0.05). All other organs and blood samples were negative for the presence of infectious virus. These data clearly demonstrate that the substitutions in BgV NS4/5 enhance viral replication in neural tissues *in vivo*. However, it was unclear which organs supported virus replication in the IP-inoculated seropositive mice, or in mice IC-inoculated with BgV_C-CG_/WNV-prME.

### Histopathological evaluation of injected mice

**Fig 6:**
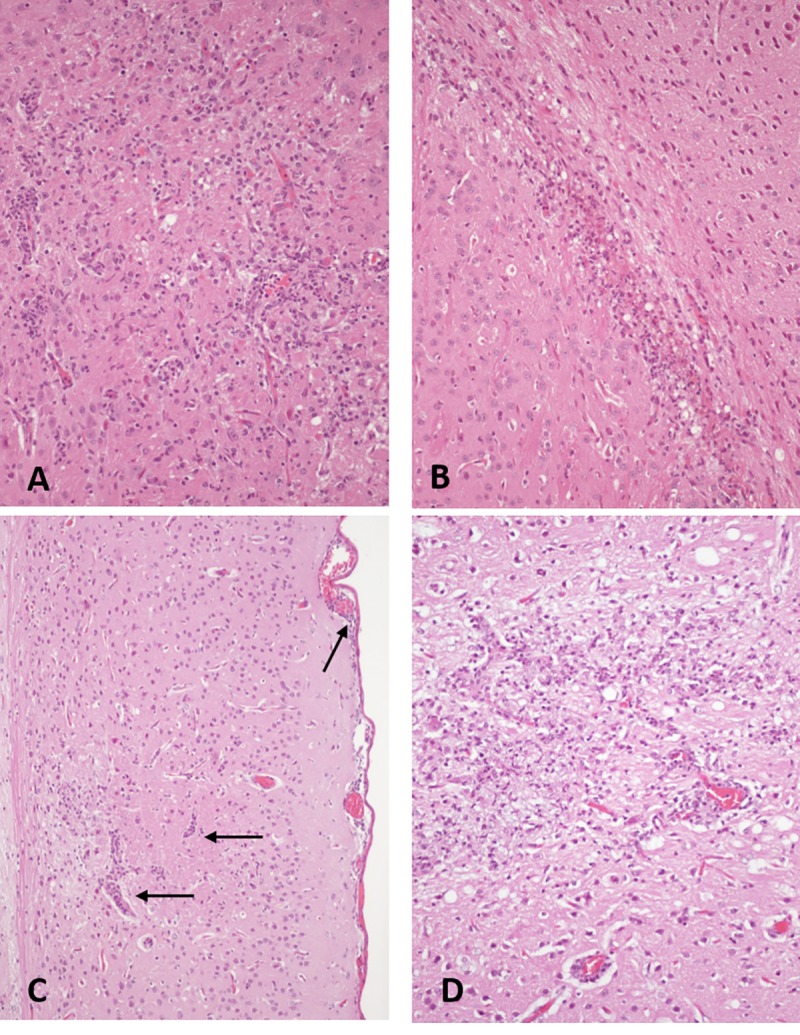
Microphotographs of meningo-encephalitis in the brains of mice IC-injected with mutant chimera BgV_T-AT_/WNV-prME. A. Brain section from mouse culled at day 3 p.i. showing widespread infiltration of neutrophils and monocytes in the neuropil accompanied by gliosis (overall marked increase in cellularity). B. Brain section from mouse culled at day 5 p.i. showing extension of inflammation into other parts of the brain distant from the injection site. C. Brain section from animal culled on day 22 p.i. where evidence of severe meningo-encephalitis is still present with perivascular cuffing and leukocyte infiltrates in the meninges (arrows). D. Brain section from mouse culled on day 22 p.i. and demonstrating persistent severe inflammation throughout the neuropil. Hematoxylin and eosin staining.

Histopathological examination of the brain from animals terminated on days three and five p.i. revealed minimal haemorrhage and neuropil spongiosis at the IC injection site in some animals. On day 3 p.i. one animal in each of the triple mutants BgV_T-AT_ and BgV_T-AT_/WNV-prME IC-inoculated groups had more widespread inflammation with perivascular cuffing, neuropil infiltration of neutrophils and monocytoid cells as well as gliosis (Fig 6A). By day 5 p.i., two out of three animals in the BgV_T-AT_/WNV-prME group had areas in the caudal putamen, thalamus and/or frontal part of *Corpus calosum* of mild to moderate infiltration of monocytes and neutrophils accompanied by gliosis, spongiosis and notable leukocyte margination in small blood vessels (Fig 6B). The brain of all other animals terminated at days three and five p.i. were microscopically normal.

Only animals in the BgV_T-AT_/WNV-prME and WNV/BgV-prME IC-inoculated groups had microscopic evidence of meningo-encephalitis on day 22 p.i.. In the BgV_T-AT_/WNV-prME group, the more severe lesions were seen in the animal that presented with mild clinical signs (Fig 6C and 6D). The animal had multifocal to coalescing neuron degeneration and segmental loss of neurons in the *Hippocampus*, vascular leukocyte cuffing and neuropil infiltration of monocyte-macrophages, gliosis and severe spongiosis, particularly in the putamen and thalamus, as well as multifocal mononuclear leukocyte infiltration of the meninges. The only two brain regions apparently spared were the olfactory bulbs and the cerebellum. The other five animals in this group, which never had any clinical signs of disease, had similar types of histopathological lesions, but generally milder and less extensive brain involvement. Six of the ten WNV/BgV-prME IC-injected animals, including the animal which had to be culled 8 days p.i., presented with histopathological evidence of meningo-encephalitis, including severe gliosis, leukocyte infiltration into the neuropil and perivascular cuffing despite lack of clinical signs in five of these mice. At none of the time points examined were any lesions detected in spinal cord, lungs, liver, heart, kidney or spleen, nor were any gross or histological changes seen in the mock infected animals. In summary, there was only clear histological evidence of pathology in mice injected with viruses that contained the three NS4/5 mutations (BgV_T-AT_ or BgV_T-AT_/WNV-prME), or contained a WNV-derived genome backbone, further supporting a role for these substitutions in enhancing viral replication and pathogenesis.

### Immunohistochemistry of organ sections

**Fig 7:**
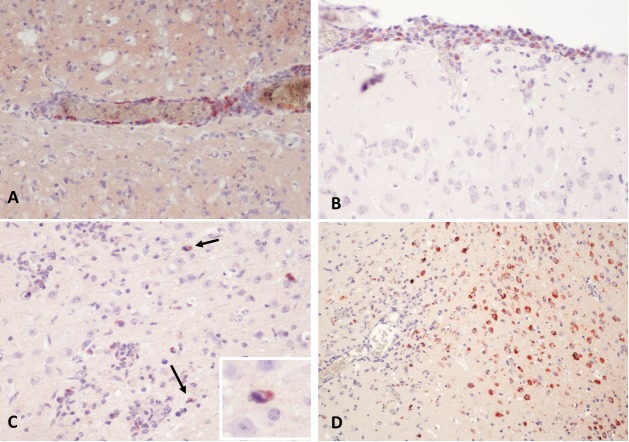
Immunohistochemistry of brain sections from mice injected with BgV-derived viruses. Virus positive cells appear with a red signal. A. Pericytes and leukocytes infiltrating in the Virchow’s space around cerebral blood vessels are positive for viral antigen in mouse culled on day 22 post-IC-inoculation of mutant chimera BgV_T-AT_/WNV-prME. B. Virus antigen positive cells amongst the leukocyte infiltrating the meninges in mouse culled on day 22 post-IC-inoculation with BgV_T-AT_/WNV-prME. C. Scattered neurons are positive for viral antigen, which is mainly located to the peri-nuclear Golgi-region (insert). Mouse culled on day 22 post-IC-inoculation of BgV_T-AT_/WNV-prME. D. Widespread cytoplasmic signal for viral antigen in brain of mouse IC-inoculated with WNV/BgV-prME and culled 8 days p.i..

We further assessed thin sections of organs harvested from inoculated mice for the presence of viral antigen by immunohistochemistry. We used either a cocktail of flavivirus E-protein specific mAbs or serum from mice infected with prototype BgV [11] for these experiments. This revealed widespread viral antigen expression in the brain of animals in the BgV_T-AT_/WNV-prME IC-inoculated group on day 22 p.i., but not in the animals from this group terminated at days three and five p.i., nor in the animals from the BgV_T-AT_ groups. The viral antigen positive cells were identified as vascular pericytes and leukocytes in the meningeal infiltrates (Fig 7A and 7B). More rarely were neurons and glial cells positive for viral antigen, and then mostly as intense labelling of the Golgi region with less intense cytoplasmic signal (Fig 7C). Other groups were not examined due to lack of evidence of viremia and pathology.

We immunohistochemically labelled brain sections from the WNV/BgV-prME IC-injected animal that had to be culled due to clinical disease on day 8 p.i., which revealed virus replication in neurons of both hemispheres (Fig 7D), similar to the virus antigen distribution seen in WNV-infected mice [18]. Viral antigens were not detected in any other tissue sampled from this animal (spinal cord, liver, spleen, lymph nodes, stomach, pancreas, small and large intestines, heart and lungs).

## Discussion

Our initial studies on BgV suggested that the virus was narrowly host-restricted in vertebrates with limited replication in vertebrate cells and in immunocompetent mice [11]. In the current study, host-restriction of BgV was defined *in vitro* and *in vivo* using a wider range of cell lines and vertebrate species that have been used by others to evaluate the host-range of arthropod-borne viruses such as Zika virus or chikungunya virus [19, 20]. We show that BgV host-restriction is temperature-dependent, with all vertebrate cell lines permissive to BgV replication at 34°C, but only a subset of these cells are permissive at 37°C. This phenotype was confirmed to be independent of any potential thermal instability of the virus particles. In addition, recent reports have revealed that the antiviral effects of IFN-I and RNAse L responses could be significantly lower at cooler temperatures than in warmer environments [21–24]. However, BgV still did not replicate at 37°C in cell lines deficient in IFN-I responses (Vero [25, 26] and MEF_IFNAR-/-_) or RNAse L (MEF_RNL-/-_), indicating that BgV host-restriction was not only due to IFN-I- or RNAse L-dependent mechanisms. Despite our data showing that the IFN-I pathways were not likely to be involved in BgV host-restriction, it should be noted that certain IFN-stimulated genes can be activated in the absence of IFN-I stimulus following flavivirus infection [27].

Temperature-dependent host-restriction in vertebrate cells has been shown to occur for another flavivirus, Rabensburg virus (RabV), which is a divergent strain of WNV [28, 29]. RabV failed to replicate in Vero, BHK, HEK293 and DF-1 cells at 37°C, as well as in house sparrows or chickens, but was capable of replicating in frog cells incubated at 28°C [30]. Similar to our findings with BgV, the investigators subsequently showed that RabV could replicate in vertebrate cells incubated at lower temperatures [2]. In a more recent study, the investigators have identified several mutations in the non-structural proteins of replication-competent variants arising as the virus was passaged in vertebrate cells at increasing temperatures [31]. However, specific viral or host factors involved in RabV temperature-dependent host-restriction have not been identified to date.

In this study, we have identified specific viral motifs associated with BgV host-restriction from BgV replication-competent variants, which arose quickly upon passaging on Vero cells (NS4A-S124F, NS4B-N244K and NS5-G2C). The direct involvement of these mutations in BgV host-restriction *in vitro* and BgV attenuation *in vivo* was confirmed using reverse engineered CPER-generated BgV clones. We demonstrated that *in vitro*, the NS4B and NS5 mutations are sufficient to restore BgV replication in Vero and DF-1 cells at 37°C while the NS4A mutation facilitates quicker replication and to higher titres. While the functional significance of these mutations is not yet clear, their position in close proximity to, but outside of the viral protease cleavage sites (NS4A/2K and NS4B/NS5) suggests a possible involvement in modulating the cleavage efficiency of BgV viral protease at these sites. Indeed, the prototype BgV sequence is sufficient to mediate efficient viral replication and presumably viral protease cleavage in all vertebrate cell lines at 34°C and a subset of cell lines at 37°C, which suggests that BgV viral protease cleavage must be significantly altered in cells such as Vero, DF-1 or MEF at 37°C. While including the two substitutions flanking the NS4B/5 cleavage site significantly enhanced BgV replication in all cell lines tested, those were only selected for in Vero cells, even in those passaged at 34°C, not in any BSR cells passage. Our data could suggest that these mutations are an essential adaptation to growth in Vero and other vertebrate cells, rather than adapting to replication at 37°C. In contrast, the NS4A substitution only occurred when BgV was passaged in Vero cells at 37°C and in conjunction with the NS4B/5 mutations. The three substitutions observed in BgV could occur in order to compensate for a restricted access to viral protease cleavage sites, due to some variation in the topology of the viral polyprotein in some cell membranes. The NS4A substitution may further optimise viral protein topology in the cell membranes, by regulating membrane remodelling as the temperature is increased to 37°C [9].

Our results were in line with previous findings that a small number of amino acid substitutions can lead to different levels of temperature-dependent attenuation. Indeed, it has been shown that a single amino acid change occurring in WNV NS4B (C102S) resulted in restricted replication in Vero cells at 41°C and a highly attenuated phenotype *in vivo* [32]. In addition, another report described a mutation in the C-terminus of WNV NS4B (E249G) which attenuated the virus as manifested by smaller plaques, lower growth kinetics and lower RNA synthesis *in vitro* as well as causing lower morbidity and mortality *in vivo* [33]. While WNV NS4B is a few amino acids longer than BgV NS4B (255 vs 247 amino acids, respectively), *in silico* analysis suggest that the two mutations (WNV E249G and BgV N244K) occur at the same position when compared in an alignment (see Fig 3C). This confirmed that single mutations in distinct residues of NS4B can be instrumental for viral replication, whether it is temperature-dependent or not. Moreover, it was shown that changes in a conserved PEPE motif just prior to the NS4A/2K cleavage site (see Fig 3C) led to drastically impaired WNV replication and virion production [34]. The BgV NS4A C-terminal sequence does not include this conserved motif, which could play a role in its host-restriction and in the advent of the compensatory NS4A mutation observed.

Our results showed that the specific residues in BgV NS4A, NS4B and NS5 identified *in vitro* were directly involved in increased infectivity and pathogenesis *in vivo*. This was revealed by comparing data from prototype-injected mice with mutant-injected mice (BgV_C-CG_ *vs* BgV_T-AT_, and BgV_C-CG_/WNV-prME *vs* BgV_T-AT_/WNV-prME). Our results also suggest that the BgV structural genes (prM and E) might be involved in BgV attenuation in vertebrates. Indeed, our results establish that while the BgV-prME proteins can facilitate infection in vertebrates, as shown by the data from WNV/BgV-prME inoculated mice, the insertion of these genes resulted in attenuated phenotypes compared to the corresponding viruses with WNV-prME. WNV/BgV-prME was significantly less virulent than WNV_FLSDX_ when compared to studies performed under similar conditions [35]–CD1 mice having received a 10^4^ IU IP dose of WNV_FLSDX_ all succumb to the infection after 8 days–and both versions of BgV seemed to replicate less efficiently than their BgV/WNV-prME counterpart in mice. Taken together, these comparisons suggest that BgV attenuation may be influenced by inefficient binding and/or entering to vertebrate cells, as well as reduced replication efficiency due to the key residues identified in the non-structural viral proteins.

The persistence of BgV_T-AT_/WNV-prME in the brains of IC-inoculated mice for 22 days, in the face of development of neutralizing antibodies is intriguing. Moreover, the viral antigen distribution observed by immunohistochemistry in these mice is similar to that seen in bovine foetuses and calves persistently infected with the non-arthropod-borne pestivirus bovine viral diarrhea virus [36, 37]. This perhaps points to a potentially very different pathogenesis in the, as yet unknown, natural vertebrate host(s) of BgV.

While components of the mechanisms involved in BgV host-restriction were elucidated in this study, it is pertinent to consider how these may be relevant to the ecology of this virus. It is possible that BgV might be amplified and horizontally transmitted by a cryptic vertebrate host in the wild, potentially a species with a lower core body temperature, based on our results showing higher levels of replication at lower temperatures in all tested vertebrate cell lines. Therefore, the lack of viral replication we observe in avian models (DF-1 cells, embryonated eggs and live sparrows), could be due to the higher core body temperature of these animals. However, no evidence was found that BgV was able to replicate efficiently in reptiles or amphibians despite their lower core body temperature. Our previous publications suggest that BgV may preferentially replicate in marsupial hosts, on which *Cx*. *annulirostris* mosquitoes prefer to feed, even though we have shown here that black-tailed wallaby cells do not seem to be particularly permissive to BgV [11, 13, 38]. Interestingly, a species of possum (Common spotted cuscus—*Spilocuscus maculatus*) present in Cape York, where BgV was isolated, maintains the lower core body temperature of 34.6°C [39]. In addition to this, potential hosts for BgV could also include animals such as monotremes, the egg-laying mammals found in Australia: platypus (*Ornithorhynchus anatinus*) or echidna (*Tachyglossus aculeatus*), which have a significantly lower core body temperature compared to other mammals [40]. This topic needs further elucidation and sampling of Australian animals sera sample could help shed some light on the natural vertebrate host of BgV.

Overall, the data presented in this report successfully identify multiple factors for the vertebrate host-restriction of a recently discovered flavivirus. This represents a significant contribution towards understanding the biodiversity of flaviviruses and the mechanisms of their host-restriction and potential for emergence as pathogens.

## Material and methods

### Ethics statement

All studies involving sparrows, frogs, toads and snakes, and conducted at Colorado State University were reviewed and approved by the Colorado State University Institutional Animal Care and Use Committee (approval 16-6934A) and were conducted under strict accordance with the Guide for the Care and Use of Laboratory Animals (8th Edition, National Research Council). All animals were euthanized via pentobarbitol overdose administered intravenously (sparrows) or intraperitoneally (reptiles and amphibians).

All procedures involving mice at the University of Queensland were approved by the University of Queensland Animal Ethics Committee (permit numbers SCMB/033/15/FIMMWA, SCMB/339/16 and SCMB/028/19), and conducted under strict accordance with the Australian code for the care and use of animals for scientific purposes (National Health and Medical Research Council). The mice were bled by cardiac puncture under ketamine/xylazine anaesthesia followed by cervical dislocation.

The chicken embryos were obtained commercially from the Darwalla group, 1 Darwalla Rd, Mt Cotton QLD 4165. The age of embryonation was ten days. The embryos were culled by freezing 45–60 minutes at -20°C.

### Cell culture

C6/36 cells (*Ae*. *albopictus*, ATCC CRL-1660) were cultured in RPMI 1640 with 2–10% FBS. HSU (*Cx*. *quinquefasciatus*, obtained from Peter Walker, Australian Animal Health Laboratory) were maintained in Leibovitz’s L-15 medium supplemented with 10% FBS and 10% tryptose phosphate broth. MOS55 (*An*. *gambiae*, obtained from Scott O’Neil, James Cook University) and S2 (*Drosophila melanogaster*, obtained from Paul Young, University of Queensland) cells were maintained in Schneider’s drosophila medium with 10% FBS. All insect cells were incubated at 28°C.

Vero (*Cercopithecus aethiops*, monkey, epithelial, ATCC CCL-81), DF-1 (*Gallus gallus*, chicken, fibroblast, obtained from Alexander Khromykh, University of Queensland), RK-13 (*Oryctolagus cuniculus*, rabbit, kidney epithelial, obtained from Timothy Mahony, Queensland Alliance for Agriculture and Food Innovation), MDBK (*Bos taurus*, cow, kidney epithelial, obtained from Timothy Mahony, Queensland Alliance for Agriculture and Food Innovation), MDCK (*Canis familiaris*, dog, kidney epithelial, ATCC CCL-34), BHK (*Mesocricetus auratus*, hamster, kidney fibroblast, ATCC CCL-10), BSR (*Mesocricetus auratus*, hamster, kidney fibroblast, obtained from Steven Davis, Berrimah Veterinary Laboratories), MEF_WT_ (*Mus musculus*, mouse, embryo fibroblast, obtained from Alexander Khromykh, University of Queensland), MEF_IFNAR-/-_ (*Mus musculus*, mouse, embryo fibroblast, IFN-I receptor knockout, obtained from Alexander Khromykh, University of Queensland), MEF_RNL-/-_ (*Mus musculus*, mouse, embryo fibroblast, RNase L knockout, obtained from Alexander Khromykh, University of Queensland) [41], SW13 (*Homo sapiens*, human, adrenal gland epithelial carcinoma, ATCC CCL-105) cells were cultured in Dulbecco’s modified Eagle’s medium (DMEM) with 2–10% FBS. HEK293 (*Homo sapiens*, human, epithelial kidney, obtained from Alexander Khromykh, University of Queensland) cells were maintained in Dulbecco’s modified Eagle’s medium (DMEM) with 2–10% FBS supplemented with 1mM sodium pyruvate and 1% HEPES. OK (*Didelphis marsupialis virginiana*, opossum, kidney epithelial, obtained from David Williams, Australian Animal Health Laboratory) cells were cultured in minimal essential media (MEM) with 1mM sodium pyruvate and 2–5% FBS. LLC PK1 (*Sus scrofa*, pig, kidney epithelial, obtained from David Williams, Australian Animal Health Laboratory) and PK15 (*Sus scrofa*, pig, kidney epithelial, obtained from David Williams, Australian Animal Health Laboratory) cells were maintained in Eagle’s minimal essential media (EMEM) with 10mM HEPES, 2–10% FBS and 2mM glutamine. ST (*Sus scrofa*, pig, testis fibroblast, obtained from David Williams, Australian Animal Health Laboratory) were maintained in EMEM with 2–8% FBS, 10mM HEPES and sodium pyruvate. JU56 (*Protomnodon bicolor*, black-tailed wallaby, buccal mucosa fibroblasts, obtained from Stacey Lynch, Victorian Department of Environment and Primary Industries) cells were maintained in Opti-MEM with 5% FBS at 37°C. A6 (*Xenopus laevis*, frog, kidney epithelial, obtained from David Williams, Australian Animal Health Laboratory) cells were maintained in 2–10% FBS RPMI. VSW (*Daboia russelii*, viper, epithelial, obtained from David Williams, Australian Animal Health Laboratory) cells were maintained in MEM with Hank’s salts, 0.35g/L sodium bicarbonate and 2–10% FBS. 3CPL (*Crocodylus porosus*, crocodile, lung fibroblast, obtained from Steven Davis, Berrimah Vet Laboratories) 3CPK (*Crocodylus porosus*, crocodile, kidney epithelial, obtained from Steven Davis, Berrimah Vet Laboratories) and 1-LV (*Crocodylus porosus*, crocodile, liver fibroblast, obtained from Steven Davis, Berrimah Vet Laboratories) cells were maintained in M199 with 25mM HEPES, Hank’s salts and 2–15% FBS [42]. All vertebrate cells were incubated at 37°C except for A6, VSW, 3CPL, 3CPK and 1-LV cells, which were incubated at 28°C. All media were supplemented with 50U penicillin/mL, 50μg streptomycin/mL and 2mM L-glutamine.

### Virus culture

The virus strains used were WNV, Kunjin strain MRM61C (WNV_KUN_) (passage unknown, Genbank accession number KX394398.1), WNV_KUN_ strain WNV_FLSDX(Pro)_ (derived from the WNV_KUN_ infectious clone FLDSX–Genbank accession number AY274504.1, with a proline substitution in residue 250 of NS1 [43]) and BgV prototype CY4270 (passage 6, passaged only in C6/36 cells, Genbank accession number KU308380 for a passage 2 stock). The WNV-derived chimeric fragments were generated from WNV_KUN_ infectious clone WNV_FLSDX(Pro)_.

### Replication in vertebrates *in vitro*

Replication in vertebrate cells was assessed in a range of cell lines (A6, 3CPK, 1LV, Vero, DF-1, RK-13, MDBK, MEF_WT_, MEF_IFNAR-/-_, MEF_RNL-/-_, JU56, VSW, 3CPL, MDCK, PK15, LLCPK1, ST, OK, BHK, BSR, HEK293 and SW13) including a C6/36 control following the method described in Colmant *et al*., 2015 [11]. Cells were inoculated with virus in triplicates in 24-well plates at a MOI 1 with 200μL of inoculum, rocked at RT for 30 minutes then incubated at 28°C or 37°C depending on the cell line for one hour. The inoculum was removed, and the cells washed three times with sterile PBS, topped up with 2% FBS growth media and incubated at 28°C or 37°C for five days. The cell culture supernatants were harvested and stored at -80°C, then titrated on C6/36 cells by TCID_50_ as described below.

### Virus titration by TCID_50_

Cell supernatants were titrated by the TCID_50_ method on C6/36 cells in 96 well plates, with 4–10 wells per 10-fold dilution [11]. The titration plates were incubated for 5 days at 28°C, cells were fixed in 20% acetone, 0.02% BSA in PBS and replication was assessed by fixed-cell ELISA (see below) with pan-flavivirus E-reactive monoclonal antibody (mAb) 4G2 [44]. The titre obtained was determined using Reed and Muench’s guidelines [45].

### Fixed-cell ELISA

Fixed cells were blocked for 30 minutes at RT in blocking buffer (0.05 M Tris/HCl (pH 8.0), 1 mM EDTA, 0.15 M NaCl, 0.05% (v/v) Tween-20, 0.2% w/v casein). Primary mAb was added to each well after removing the blocking buffer and incubated at 37°C for one hour. Plates were washed with PBS containing 0.05% Tween-20 (PBS-T) four times and secondary HRP-conjugated antibody (goat anti-mouse, Dako) was added at the optimal concentration in blocking buffer and incubated at 37°C for one hour. Plates were washed six times with PBS-T and ABTS based substrate (1mM 2,2'-azino-bis(3-ethylbenzothiazoline-6-sulphonic acid)) with 3mM hydrogen peroxide in a 0.1M citrate / 0.2M Na_2_PO_4_ buffer pH 4.2) was added and left to develop in the dark at RT for one hour. Finally, the absorbance of each well was measured by an automated 96-well spectrophotometer at 405 nm.

### Infections in embryonated chicken eggs, toads, frogs, birds and snakes

The growth of virus in embryonated chicken eggs was assessed using a modification of a previously published method [46]. Briefly, 50μL of diluted virus were inoculated intravenously into three groups of three 9–12 days old embryonated chicken eggs with the following doses: 10^7^ TCID_50_ IU/mL, 10^6^ TCID_50_ IU/mL and 10^5^ TCID_50_ IU/mL (*i*.*e*. 10^5.7^, 10^4.7^ and 10^3.7^ TCID_50_ IU/egg, respectively). Embryonated chicken eggs are used for arbovirus isolation [47], but they are not routinely used in host-restriction experiments so the inoculum doses were chosen arbitrarily: an undiluted virus stock was used alongside two diluted doses, 1:10 and 1:100. The rationale was to use as much virus as possible in order to facilitate infection and to avoid the effects of interfering particles should they occur. The eggs were incubated at 33–35°C and candled daily. Embryos that died between days 2 and 5 were retained at 4°C and embryos remaining alive at 5 days post infection were culled. Whole embryos were homogenised after removal of their heads in approximately 10 mL of heart-brain-broth, after pooling the embryos inoculated with the same dose and culled at 5 days p.i.. The debris was removed by centrifugation and the resulting approximate 5 mL of clarified egg homogenates were titrated by TCID_50_ on C6/36 cells as described above, incubated five days at 28°C and analysed by fixed-cell ELISA.

Five house sparrows (*Passer domesticus*), six northern leopard frogs (*Lithobates pipiens*), six Texas toads (*Bufo spp*.), and six ribbon snakes (*Thamnophis spp*.) were inoculated subcutaneously with 0.1 ml containing 1x10^6^ TCID_50_ IU of BgV diluted in PBS, under Colorado State University animal ethics approval number 16-6934A. The dose chosen was higher than would be expected from the bite of a single infected mosquito, in order to have confidence that if negative results were obtained it was not due to an inadequate inoculum but also to remain realistic. This dose was similar to the one used for Zika virus in other host-restriction studies in these animals [48]. Blood samples were collected from sparrows daily on days 1–5 and on day 14 post-inoculation. Blood was collected from frogs, toads and snakes on days 1, 3, 5, 7 and 28 post-inoculation. Blood samples were diluted 1:5 in BA1 medium (HEPES-buffered MEM containing 1% BSA), centrifuged to remove cells, and stored at -80°C until assay. Infectious virus was assayed from all samples by inoculation of duplicate 0.1 ml aliquots of samples diluted 1:5 and 1:50, along with a BgV positive control, onto monolayers of C6/36 cells in 96 well plates. Two days later, the medium was decanted and cells fixed with 80% acetone. Virus infection of cells was assessed after immunostaining with 4G2 antibody and DyLight-conjugated anti-mouse IgG (Jackson ImmunoResearch, West Grove, PA, USA) [49].

### Infections at lower temperatures

A subset of cell lines (Vero, DF-1, MEF_WT_, MEF_IFNAR-/-_, MEF_RNL-/-_, JU56, HEK293, LLCPK1, PK15A, ST, OK, SW13, BSR) were selected to be infected with BgV at the lower temperature of 34°C. These infections were performed similarly to what has been described above, at 34°C and 37°C in parallel, in triplicates, with WNV_KUN_ as replicating virus control and with C6/36 cells as permissive cells control. Supernatants from these infected vertebrate cells were titrated on C6/36 cells by TCID_50_ as described above and analysed in fixed-cell ELISA using 4G2. The data were plotted with average and standard deviation of the triplicate values for each sample.

### Thermostability assay

Triplicates of 1x10^5^ infectious particles of virus were incubated in cell culture medium with 2% FBS at 4°C, 28°C, 34°C and 37°C and harvested at 30min, 24h, 48h and 72h after the start of incubation. The harvested virus was stored at -80°C and titrated on C6/36 cells as described above. Thermostability graphs were plotted in GraphPad Prism 7 with average and standard deviation of the triplicate values for each sample.

### Threshold of temperature for BgV infectivity

BSR and Vero cells were infected at MOI 1 with a stock of C6/36-derived BgV in T25 flasks and incubated at 34°C, 35°C, 36°C or 37°C to determine a threshold of temperature allowing for BgV replication. The supernatant of these cells was harvested and stored at -80°C before being titrated by TCID_50_ on C6/36 cells, incubated at 28°C for five days, fixed in 20% acetone, 0.02% BSA in PBS and analysed by fixed-cell ELISA using 4G2.

### Sequencing of untranslated regions

As the terminal UTR sequences could not be determined previously by next generation sequencing [11], the viral RNA was circularised following the method adapted by Khromykh *et al*. [15, 16]. Briefly, BgV supernatant from two T175 flasks of C6/36 infected cells was PEG precipitated by adding one part of 40% PEG8000 in TNE (12 mM Tris at pH 8, 120 mM NaCl, 1 mM EDTA pH8) to four parts of infected cell culture supernatant and spinning on a rotary shaker overnight and centrifuging for one hour at 21,612 g (12,000 rpm; JLA 16.250 rotor Beckman) at 4°C. The precipitated virus pellet was resuspended in 500μL sterile PBS. RNA was extracted from the purified virions using the Macherey Nagel RNA isolation kit following the manufacturer’s instructions. RNA extracts were quantified using Nanodrop (Biolab ND-1000 Spectrophotometer). One microgram of RNA was decapped with tobacco acid pyrophosphatase at 37°C for one hour and circularised with T4 RNA ligase at 4°C overnight. The circularised RNA was reverse transcribed to cDNA with Superscript III (Thermo-Fisher Scientific) with a BgV reverse primer specific for the prM gene (see S1 Table). A PCR product containing the joined UTRs was amplified with Phusion High Fidelity DNA polymerase (NEB) in a high-fidelity buffer and Sanger sequenced by the Australian Genome Research Facility (AGRF, Brisbane). The junction site of the end of the 3’ UTR and the start of the 5’ UTR was determined based on consensus established from alignment of flavivirus UTR sequences and confirmed by reverse transcription PCR (RT-PCR) using primers specific for these ends (see S1 Table) and SuperScript III One-Step RT-PCR System with Platinum Taq DNA Polymerase (Invitrogen).

### Infectious BgV DNA construct and chimeras

An infectious clone of BgV and two chimeric DNA infectious constructs were generated using CPER as described previously, with primers listed in S1 Table, under the Institutional Biosafety Subcommittee number IBC/1178/SCMB/2018 [17]. The chimeras created were WNV/BgV-prME containing a WNV_FLSDX(Pro)_ genome backbone with the BgV prME genes and BgV/WNV-prME containing a BgV genome backbone and WNV_FLSDX(Pro)_ prME genes.

The genome of WNV/BgV-prME was sequenced with the following protocol. Genomic RNA of the chimeric virus was converted into cDNA using specific primers at the 3’ end of the 3’UTR and Superscript IV reverse transcriptase (Thermo-Fisher Scientific) following the manufacturer’s protocol (see S1 Table). Fragments were amplified using Q5 High Fidelity DNA polymerase (NEB) following manufacturer’s protocol and using virus specific primers (S1 Table). The fragments were combined in equimolar amounts, quantified and sequenced at the Australian Centre for Ecogenomics (University of Queensland, Brisbane, Australia). Libraries were prepared with the Nextera XT DNA Sample Preparation kit (Illumina Inc., San Diego, CA). The prepared library was sequenced on an Illumina NextSeq500 platform 2*150 bp PE run with V2 chemistry. Reads were mapped to the chimera genome in Geneious 8.1.9 with default parameters.

The genome of BgV/WNV-prME was sequenced as follows. RNA was treated with Heat&Run DNase (ArcticZymes) to remove contaminating host DNA and the DNAse was heat inactivated at 80°C for five minutes. First strand cDNA was generated using Protoscript II (New England Biolabs), using supplied random primer mix and reaction conditions recommended by the manufacturer. The reaction product was then converted to double-stranded DNA (16°C for 60 min) using an enzyme mixture consisting of E. coli DNA ligase, DNA polymerase I and RNase H (New England Biolabs), followed by heat inactivation (80°C for 5 min). The double-stranded cDNA was used a template for library construction using the Nextera XT library kit (Illumina) with barcoded primers. The library was sequenced on a NextSeq 500 generating 2 x 151 bp paired reads.

### Replication kinetics

Cells were seeded at 2x10^5^ cells per well of a 24 well plate, in triplicate wells for each virus at each time point and each temperature. Cells were inoculated with virus at a MOI of 0.1 with 500μL of virus, incubated at 28/34/37°C for 1h 30min. The inoculum was removed, and the cells washed three times with sterile PBS, topped up with 2% FBS growth media and incubated at 28/34/37°C for five days. Supernatant was harvested at each time point from the dedicated well and stored at -80°C. The supernatants were titrated on C6/36 cells and TCID_50_ titres were determined by fixed-cell ELISA as described above. Replication kinetics curves were plotted with average and standard deviation of the triplicate values for each sample.

### Immunofluorescence assay

Coverslips were incubated for one hour at RT with blocking buffer (see above). Primary mAb at the optimal dilution in blocking buffer was added to each coverslip after removing the blocking buffer and incubated rocking at RT for one hour. Coverslips were washed with PBS-T three times and secondary antibody (Alexafluor 488-conjugated goat anti-mouse IgG H+L, Invitrogen) was added at the optimal concentration in blocking buffer and incubated rocking at RT in the dark for one hour. Secondary antibody was removed and Hoechst 33342 nuclear stain (Invitrogen) was added at the recommended concentration and incubated for five minutes in the dark, rocking at RT. Coverslips were washed twice with PBS-T and once with PBS and mounted on microscope slides with a drop of ProlongGold antifade (Invitrogen). Fluorescence was observed and imaged on a Zeiss microscope at a 400 or 200 times magnification. Post-processing of the raw images was performed using Microsoft Office Picture Manager (2010) and ImageJ.

### Passaging in vertebrate cells at increasing temperatures

BSR and Vero cells were infected with a stock of BgV generated in C6/36 cells at an MOI of 1 in T25 flasks and directly incubated at 34°C for five days, without removing the inoculum (passage 1 –P1). The P1 Vero cells supernatant was blind-passaged by transferring 1mL to three freshly seeded T25 flasks of Vero cells incubated at 34°C, 35°C and 37°C, respectively, for six days (P2 supernatant). The P1 Vero cells were split by removing cell culture supernatant, washing the monolayer with 10mL of sterile PBS and trypsinizing at 37°C for five minutes. The cells were resuspended in 10mL cell culture medium and 1mL was seeded in three T25 flasks incubated at 34°C, 35°C and 37°C respectively for six days (P2 cells). The P1 BSR cells supernatant was similarly blind-passaged on three T25 flasks incubated at 34°C, 35°C and 37°C, respectively, for six days (P2 supernatant). The BSR cells succumbed to strong cytopathic effect from the infection and could not be passaged. This process was repeated as indicated in Fig 3A (P1-4), by passaging supernatant and cells either at stable temperatures (34°C or 37°C) or at increasing temperatures (35°C to 36°C to 37°C). At each passage, an aliquot of cell culture supernatant was harvested and stored at -80°C. After all the passages were completed, all samples were titrated by TCID_50_ on C6/36 cells, incubated at 28°C for five days, fixed in 20% acetone, 0.02% BSA in PBS and analysed by fixed-cell ELISA using 4G2.

Selected samples were further passaged (P5, circled in Fig 3A) in order to confirm replication of BgV. For that purpose, 200μL of P4 supernatant were inoculated on both BSR and Vero cells seeded in a 24 well plate and incubated one hour at 37°C. The inoculum was removed, the cells were washed thrice with sterile PBS, replenished with 1mL of cell culture medium and incubated for five days at 37°C. Similarly to the previous samples, these cell culture supernatants were titrated by TCID_50_ on C6/36 cells, incubated at 28°C for 5 days, and analysed by fixed-cell ELISA using 4G2.

### Sequencing of mutated BgV

RNA was extracted from selected samples from P5 of the passaging experiment and from the original C6/36-derived BgV stock using the Macherey Nagel Nucleospin RNA Virus isolation kit, following the manufacturer’s protocol–without carrier RNA. This RNA was sequenced as described in the earlier section detailing the sequencing of BgV/WNV-prME on a NextSeg 500 (see above). The reads were assembled to the published sequence of BgV (KU308380.1) using Geneious 8.1.9 with default parameters.

RNA was extracted from all other BgV positive supernatants using the same kit following manufacturer’s instructions. The mutation sites were amplified by RT-PCR using SuperScript III One-Step RT-PCR System with Platinum Taq DNA Polymerase (Invitrogen) and BgV specific primers (see S1 Table). The resulting PCR products were resolved on a 1% agarose gel, excised and gel purified using Nucleospin gel and PCR clean up kit (Macherey Nagel), before they were sent for Sanger sequencing at the AGRF. The sequences were aligned to a BgV reference and each other in Geneious 8.1.9.

### Infectious mutant BgV DNA constructs

Three mutated versions of BgV infectious clone (BgV_T-CG_, BgV_C-AT_ and BgV_T-AT_) as well as a mutated version of the BgV/WNV-prME chimera (BgV_T-AT_/WNV-prME) were generated using CPER as described above, alongside non-mutated versions (BgV_C-CG_ and BgV_C-CG_/WNV-prME), under the Institutional Biosafety Subcommittee number IBC/1178/SCMB/2018. The mutations were introduced using specifically designed primers (see S1 Table). Successful production of the mutants and infectious clone were confirmed by IFA with 4G2 on P0 transfected cells as well as later passages, and by TCID_50_ of P0 transfected and P1 infected cell supernatants. RNA was extracted from the P1 supernatant of all four viruses produced and the mutation sites amplified by RT-PCR as described above and Sanger sequenced at AGRF as described above to confirm their authenticity.

### Mouse infections

All mouse experiments were performed at the University of Queenlsand in accordance with the ethical standards of the Australian Code for the Care and Use of Animals for Scientific Purposes and were approved by the University of Queensland Animal Ethics Committee before commencement of the studies (permit numbers SCMB/033/15/FIMMWA, SCMB/339/16 and SCMB/028/19). To assess the ability of BgV-derived viruses to infect, replicate and produce disease in young mice, six groups of ten mice (male and female), 19 days old at start of experiment, were challenged with 10^4^ TCID_50_ IU of BgV_Clone_, and the chimeric viruses BgV/WNV-prME and WNV/BgV-prME or mock-infected *via* either the IP or IC route. CD1 mice were used as they have an intact immune response, are outbred, and have been shown to be susceptible to flavivirus infection at these doses [50]. One mouse from the BgV_Clone_ IC inoculated group succumbed to the anaesthesia. The mice were observed twice daily until day 8 post-inoculation and daily after that, until termination at day 21 post-inoculation. A second cohort was inoculated with BgV-derived viruses. Groups of twelve mice (six male and six female) were infected *via* either the IP or IC route with 10^4^ TCID_50_ IU of BgV_C-CG_, BgV_T-AT_, BgV_C-CG_/WNV-prME or BgV_T-AT_/WNV-prME. Four mice served as mock-infected controls (two mice per route). Three mice per group were terminated on days three and five p.i. and subjected to complete necropsy, blood and tissue sampling for virus isolation and histopathology (see below). The remaining six mice per group were culled on day 22 p.i. and similarly subjected to blood and tissue sampling. One BgV_C-CG_ IC-inoculated mouse succumbed to the anaesthesia. The mice were observed twice daily until day 13 p.i. and daily after that, until termination at day 22 p.i.. All mice were bled by cardiac puncture under ketamine/xylazine anaesthesia followed by cervical dislocation on day 21–22 p.i..

### Seroconversion and microneutralisation

Heat-inactivated sera from all 59 mice were tested for seroconversion and cross-reactivity by fixed-cell ELISA as described above, on fixed monolayers infected with prototype BgV, WNV_KUN_, BgV/WNV-prME and WNV/BgV-prME. The sera were diluted from 1/20 to 1/160 in blocking buffer by doubling dilutions. These data were not analysed for statistical significance, as in all cases, the two groups to be compared had either very low numbers of positive replicates, or were all equal to the higher limit of detection, skewing the analysis.

Virus neutralisation by the sera was also assessed for BgV and WNV_KUN_. Sera were diluted from 1/20 to 1/2560 by two-fold dilutions in growth medium. Fifty microliters of each dilution of sera were incubated in duplicate in 96 well plates at 37°C for one hour with 50μL of virus diluted to 100 TCID_50_ IU per well. C6/36 cells were then added (2x10^4^ per well) and the plates incubated at 28°C for five days. Neutralisation of the viruses was analysed by fixed-cell ELISA with 4G2 as described above. The neutralisation titre was determined as the highest dilution at which all virus replication was inhibited. The data were statistically analysed using an unpaired parametric t-test and the only two groups that could be analysed (IC-inoculated BgV_T-AT_ and BgV_T-AT_/WNV-prME) were not statistically significantly different.

### Tissue processing for virus isolation

Each tissue destined to virus isolation (half spleen, one kidney, half liver and one brain hemisphere) was harvested sterile and snap frozen on dry ice, while the collected blood was kept on wet ice. The organs were weighed and 20% weight/volume homogenates were generated using 2% FBS RPMI with PSG, two glass beads and a Tissue Lyser III (Qiagen) set to 30Hz for five minutes. The mesenteric lymph node homogenates were generated using 1mL of media for all samples. The homogenates were centrifuged at 11,000g for ten minutes to pellet the solid fraction. The supernatant was then titrated on C6/36 cells as per the TCID_50_ method described above. The collected blood samples were centrifuged at 11,000g for ten minutes in Becton-Dickinson microtube with Microgard SST gold to separate the red blood cells and the serum. The serum was titrated as described above by TCID_50_ on C6/36 cells.

### Histopathology and immunohistochemistry

The organs harvested for histopathology and immunohistochemistry were fixed in 10% buffered formalin and processed for histopathology and immunohistochemistry (IHC) as previously described [18]. Heads were collected from the group injected IC with WNV/BgV-prME, while head, spine, heart, lungs, spleen, liver, and kidney were harvested from mice inoculated with BgV_C-CG_, BgV_T-AT_, BgV_C-CG_/WNV-prME and BgV_T-AT_/WNV-prME, on days 3, 5 and 22 p.i..

### Analyses

The titres obtained by TCID_50_ were determined by Reed and Muench’s guidelines [45]. Unless stated otherwise, data were statistically compared using two-way ANOVA, with Tukey’s test as correction. The cut-off *p* value used for significance was *p* < 0.05. All analyses were conducted using Graphpad Prism Version 7 (GraphPad Software, Inc, San Diego, USA).

## Supporting information

S1 Fig Replication kinetics of BgV_C-CG_ (prototype) and BgV_T-CG_ (single mutant NS4A) in C6/36 (28°C), BSR and Vero cells (37°C). The cells were inoculated in triplicates at MOI 0.1 as above, supernatants were harvested at 2, 24, 48, 96 and 120h for C6/36 cells and at 2, 24, 72 and 120 hours p.i. for vertebrate cells, stored at -80°C, titrated on C6/36 cells and the titres determined by fixed-cell ELISA. Error bars represent the standard deviation and the dotted lines represent the lower limit of detection.
(TIF)

S1 Table Primer sequences. (TIF)
