# Supplementary figures and images for: NS4/5 mutations enhance flavivirus Bamaga virus infectivity and pathogenicity in vitro and in vivo
Source: PLoS Negl Trop Dis. 2020 Mar 23;14(3):e0008166. doi: 10.1371/journal.pntd.0008166 (PMC7089401; doi:10.1371/journal.pntd.0008166)

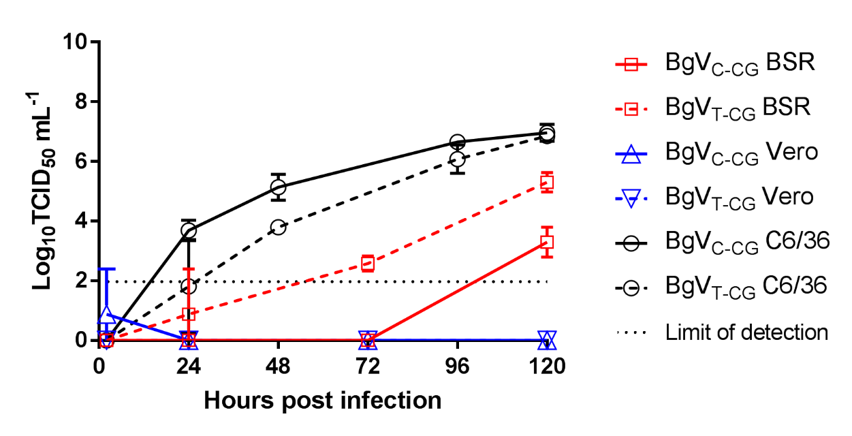

Supplement: S1 Fig — The cells were inoculated in triplicates at MOI 0.1 as above, supernatants were harvested at 2, 24, 48, 96 and 120h for C6/36 cells and at 2, 24, 72 and 120 hours p.i. for vertebrate cells, stored at -80°C, titrated on C6/36 cells and the titres determined by fixed-cell ELISA. Error bars represent the standard deviation and the dotted lines represent the lower limit of detection. (TIF) [file pntd.0008166.s001.tif]

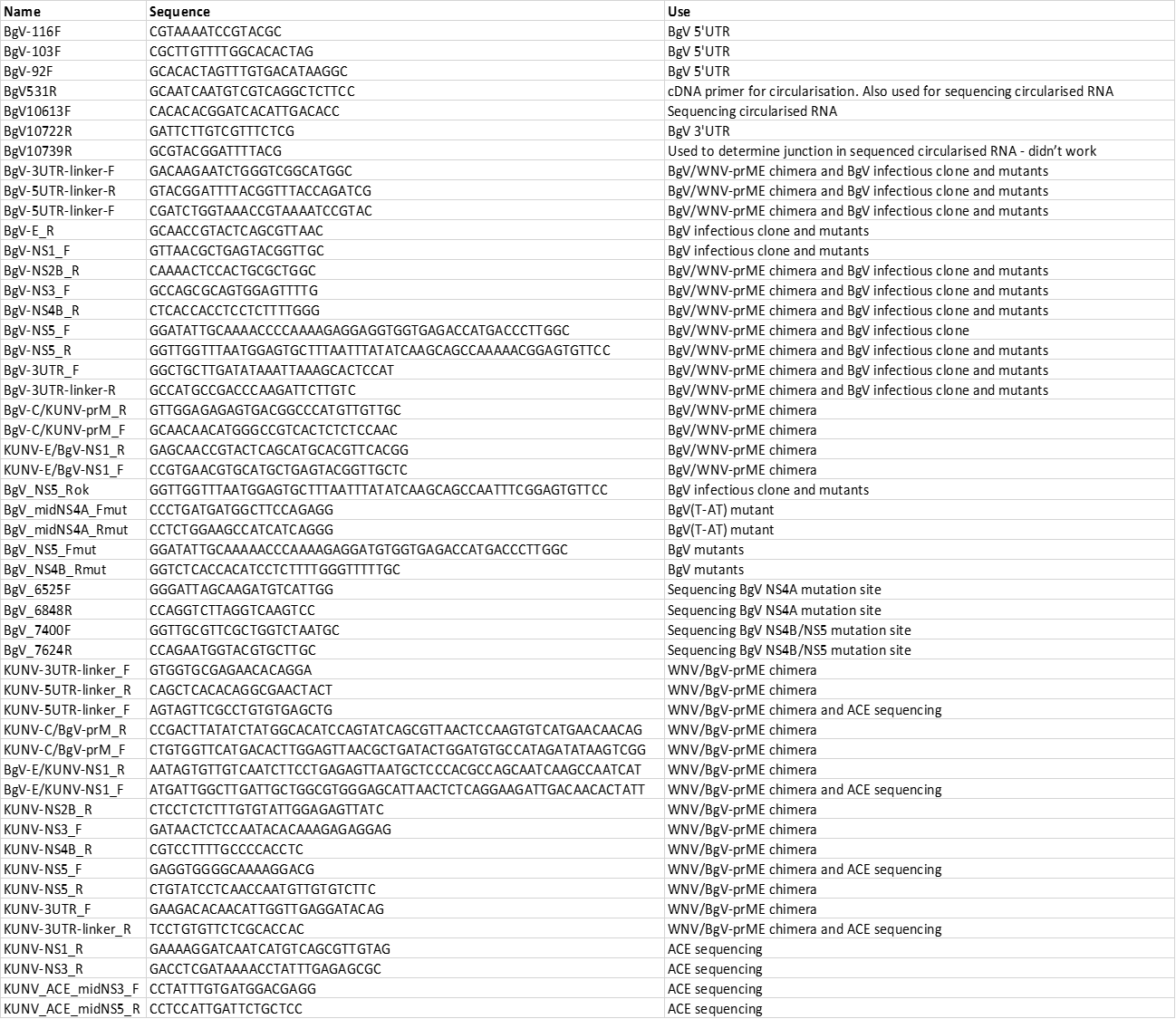

Supplement: S1 Table — (TIF) [file pntd.0008166.s002.tif]
